# Controlling angular dispersions in optical metasurfaces

**DOI:** 10.1038/s41377-020-0313-0

**Published:** 2020-05-06

**Authors:** Xiyue Zhang, Qi Li, Feifei Liu, Meng Qiu, Shulin Sun, Qiong He, Lei Zhou

**Affiliations:** 1 State Key Laboratory of Surface Physics, Key Laboratory of Micro and Nano Photonic Structures (Ministry of Education), and Department of Physics, Fudan University, Shanghai, 200438 China; 2 Shanghai Engineering Research Center of Ultra-Precision Optical Manufacturing, Green Photonics and Department of Optical Science and Engineering, Fudan University, Shanghai, 200433 China; 3 Academy for Engineering and Technology, Fudan University, Shanghai, 200433 China; 4 Collaborative Innovation Center of Advanced Microstructures, Nanjing, 210093 China

## Abstract

Although metasurfaces have shown great potential for manipulating light, most previously realized meta-devices suffer from *uncontrolled* angular dispersions, making them unfavorable for many applications. Here, we propose a general strategy to realize optical metasurfaces with desired angular dispersions based on carefully controlling both the near-field couplings between meta-atoms and the radiation pattern of a single meta-atom. Utilizing such a strategy, we experimentally demonstrate a series of optical meta-devices with predesigned angular dispersions, including two incident-angle-*insensitive* absorbers, one incident-angle-*selective* absorber, and one multifunctional meta-polarizer whose functionality changes from a perfect mirror to a half-waveplate as the excitation angle varies. Finally, we design a *gradient* meta-device using meta-atom arrays with purposely controlled angular dispersions and numerically demonstrate that it can exhibit distinct wavefront-control functionalities when illuminated at different incident angles. Our findings establish a new platform for achieving angle-multiplexed functional meta-devices, significantly expanding the wave-manipulation capabilities of optical metasurfaces.

## Metasurfaces: Controlling interactions with angular light

Scientists can control how optical meta-surfaces interact with different angles of incident light by making changes to their individual building blocks. The finding, by Lei Zhou and colleagues at China’s Fudan University, could lead to the realization of angle-multiplexed meta-devices. The researchers measured how light was reflected by a typical optical meta-surface depending on its incident angle. Their observations led them to theorize that reflected light was controlled by the interactions between meta-atoms and by radiation from individual ones. Changes to the meta-atom arrays allowed them to fabricate devices that altered light’s polarization, selectively absorbed light based on the incident angle, or absorbed light regardless of it. Finally, they were able to simulate a device with specific meta-atom arrangements that could both focus light or act as a mirror depending on the incident angle.

## Introduction

Controlling light at will is a key aim in optics research and is the basis for optical applications. Conventional optical devices, made from natural materials, are usually bulky in size (in terms of wavelength) and/or of curved shape, which is unfavorable for modern integration-optics applications^1^. Metasurfaces, ultrathin metamaterials constructed by subwavelength planar microstructures (e.g., “meta-atoms”) with tailored optical responses, have recently shown extraordinary capabilities to manipulate light in predesigned manners^2–6^. Many fascinating effects have been demonstrated based on *periodic* or *inhomogeneous* metasurfaces, such as polarization control^7–10^, perfect absorption^11–13^, light bending^14–16^, surface wave coupling^17–19^, meta-lensing^20–23^, meta-holography^24–30^, and many others^31–39^. Relying on abrupt phase changes at device surfaces rather than the propagating phases of light inside the systems, these meta-devices can be ultrathin and flat, which are highly desired in integration-optics applications.

Despite the impressive success already achieved for metasurfaces, however, most of those light-manipulation effects were only demonstrated under normal-incidence excitation, and the *angular dispersions* of the devices were often overlooked. In reality, however, angular dispersion is a critical issue that must be carefully addressed in different application scenarios. For example, while incident-angle-*insensitive* perfect absorbers are highly desired in energy-related applications, incident-angle-*selective* meta-devices have great potential in sensor-related applications. Unfortunately, the meta-devices realized thus far usually exhibit *uncontrolled* angular dispersions, which were only known after (rather than before) the devices were designed. Although a few attempts recently appear to have achieved wide-angle meta-devices^29,30^ and angle-multiplexed meta-devices^27,34–37^, the designs were typically obtained through brute-force simulation, an approach not generic enough to be applied to other cases. Very recently, we theoretically revealed that the *angular dispersions* in metasurfaces are dictated by the near-field couplings (NFCs) among adjacent meta-atoms in such systems, but the theory only considered frequency shifts, and the experimental demonstrations were limited to low-frequency domains (e.g., the THz regime)^40^.

**Fig. 1:**
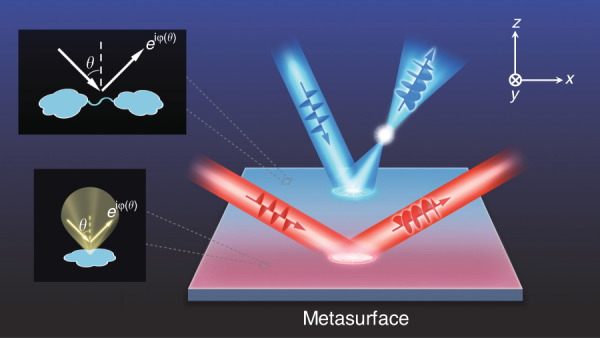
Physical origins of angular dispersions and schematics of angle-dependent multifunctional meta-devices. By controlling the coupling between meta-atoms (upper inset) and the radiation properties of constituent meta-atoms (lower inset), one can realize angle-dependent multifunctional meta-devices with wave-control functionalities that change as the incident angle of the excitation light varies

In this article, we establish a *general* and *systematic* strategy to guide the design of optical metasurfaces with *fully controlled* angular dispersions. Specifically, we show that the angular dispersions of metasurfaces are determined by both the NFCs between meta-atoms and the radiation pattern of a single constituent meta-atom (see the insets in Fig. 1). Based on such a complete theory, we experimentally demonstrate three sets of *periodic* meta-devices working in the near-infrared (NIR) regime with predesigned angular dispersions, including two incident-angle-*insensitive* perfect absorbers, an incident-angle-*selective* absorber, and an incident-angle-dependent multifunctional polarization controller (see Fig. 1). We finally employ such a generic strategy to design an angle-multiplexed wavefront-control meta-device and numerically demonstrate its bifunctional performances (e.g., focusing and acting as a mirror, see Fig. 1) when illuminated at different incident angles. Our findings pave the way to realizing meta-devices with fully controlled angular dispersions, which significantly expand the capabilities of metasurfaces to manipulate light and can further stimulate realization of high-performance optical meta-devices for versatile applications in different scenarios.

## Results

### Revealing the origins of angular dispersions in metasurfaces

**Fig. 2:**
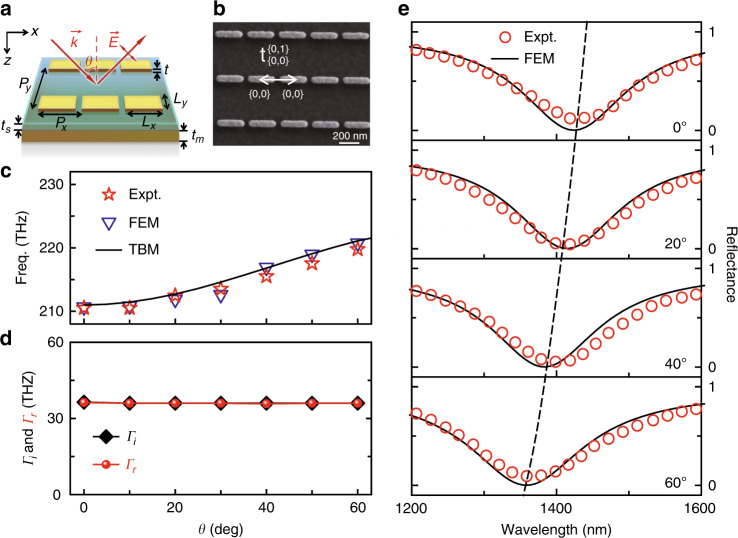
Angular dispersions of a typical optical metasurface. **a** Schematic of a perfect meta-absorber in the MIM configuration under illumination by TM-polarized light. **b** Top-view SEM image of the fabricated sample, with $t_{(0,0)}^{(0,1)}$ denoting the coupling strength between the two meta-atoms labeled {0,0} and {0,1}. **c** Resonant frequency of the meta-absorber as a function of incident angle, obtained by experiments, FEM simulations and TBM calculations. **d** Damping parameters $\Gamma _i$ and $\Gamma _r$ of the meta-absorber as functions of the incident angle, retrieved from FEM simulations. **e** Measured and simulated reflectance spectra of the meta-absorber illuminated by TM-polarized light at different incident angles. The geometrical parameters of the meta-surface are *P*_*x*_ = 270 nm, *P*_*y*_ = 400 nm, *L*_*x*_ = 240 nm, *L*_*y*_ = 50 nm, *t* = 30nm, *t*_*s*_ = 45 nm and *t*_*m*_ = 150 nm

We start by experimentally illustrating the angular responses of a typical optical metasurface. As schematically shown in Fig. 2a, the device is in a metal–insulator–metal (MIM) configuration, which consists of a periodic array of gold patch resonators and an optically thick continuous gold film separated by a dielectric (SiO_2_) spacer. We carefully design the device^11–13^ so that it can perfectly absorb incident light at a particular NIR wavelength under *normal* incidence. We fabricate a sample based on our design (see Fig. 2b for an image of the sample), and then experimentally characterize its reflection properties under illumination by light with transverse magnetic (TM) polarization at different incident angles (see Fig. S1 for the experimental setup). All measured reflected signals are normalized against a reference obtained under the same conditions but with the sample replaced by a gold mirror. Figure 2e depicts the measured reflectance spectra for different incident angles, which are in excellent agreement with the corresponding finite-element-method (FEM) simulations (see Fig. S2 in the Supplementary Information for more reflectance spectra). While each reflectance spectrum exhibits a well-defined resonance dip corresponding to (nearly) perfect light absorption, the working frequency of the device undergoes an obvious blueshift as the excitation light incident angle increases, manifesting a typical angular-dispersion behavior that was also discovered in previous studies^13,41^.

We now theoretically explain these experimental results, starting from the angle-dependent resonance frequency shift. According to the photonic tight-binding method (TBM) established for periodic metasurfaces^40^, the resonant mode of a given metasurface “seen” at an off-normal incident angle *θ* is essentially a Bloch mode with parallel **k** vector matching that of the incident wave, and thus, its frequency *f*_*r*_(*θ*) is determined by the following dispersion relation:

1 $$f_r\left( \theta \right) = f_0 + J_0 + J_1{\mathrm{cos}}\left( {Pk_0\sin \theta } \right) + J_2\cos \left( {2Pk_0\sin \theta } \right) + \,...$$

where *f*_0_ is the frequency of the resonance mode supported by a single MIM meta-atom, {*J*_*i*_} denote the couplings between meta-atoms, *P* is a lattice constant, and *k*_0_ = *ω*/*c* is the free-space wavevector. Specifically, *J*_0_, *J*_1_, and *J*_2_ describe the effective couplings within each meta-atom row, between two nearest-neighbor rows, and between next-nearest-neighbor rows, respectively. As discussed in ref. ^40^, the parameters {*J*_i_} are determined by the coupling strength between two meta-atoms located at two different lattice positions, which can be *quantitatively* calculated by the TBM when the EM fields of the single resonant mode are known^42^ (see Section 3 in Supplementary Information for more details). In addition, since the TBM can be employed to accurately predict the **k** dependences of the resonance modes supported by a metasurface/metamaterial, one can further combine the TBM with an effective-medium theory^43,44^ to study the nonlocal responses (i.e., *ε*(**k**) and *μ*(**k**))^38,39^ of the meta-system, which are closely related to the angular dispersion behavior discussed here.

We employ the photonic TBM to *quantitatively* compute all necessary inter-meta-atom coupling constants and find that the intra- and inter-row coupling strengths for this particular system are $\left\{ {J_0{\mathrm{ = 4}}{\mathrm{.53}}\,{\mathrm{THz}},\,J_{\mathrm{1}}{\mathrm{ = - 20}}{\mathrm{.14}}\,{\mathrm{THz}},\,J_{\mathrm{2}}{\mathrm{ = - 3}}{\mathrm{.05}}\,{\mathrm{THz}}...} \right\}$. Inputting these parameters into Eq. (1), we then analytically calculate the dispersion relation *f*_*r*_(*θ*) and depict it as a black solid line in Fig. 2c. The TBM results are in excellent agreement with both FEM simulations (blue triangles) and experimental results (red stars). Moreover, we can establish a clear picture to explain the blueshift of the resonant frequency with increasing *θ* based on Eq. (1). Obviously, the nearest-neighbor inter-row coupling *J*_1_ is the most important parameter in generating the angular dispersions (see Eq. (1)), while its negative sign directly dictates the blueshift of the resonance frequency.

We further theoretically explain the angle dependence of the optical line shapes (e.g., the reflectance spectra) of the metasurface. Following the coupled-mode-theory (CMT) analyses presented in ref. ^45,46^, we can describe such a system as a one-port single-mode model and derive its reflection coefficient as

2 $${r} = - {1} + \frac{{2 \ast \Gamma _{r}}}{{{\mathrm{ - i2}}\pi \left( {f - f_r} \right) + \Gamma _{\mathrm{i}} + \Gamma _r}}$$

where $\Gamma _i$ and $\Gamma _{\mathrm{r}}$ denote the damping rates of the resonance mode due to absorption loss and radiation loss, respectively. In principle, all model parameters in the above expression (i.e., ${\mathrm{f}}_r,\,\Gamma _{\mathrm{i}},\, \Gamma _r$) exhibit different dependences on the incident angle *θ*, which collectively dictate the angular dispersion of the whole response. With the ${\mathrm{f}}_r\sim \theta$ relation fully determined (see Eq. (1)), we can further retrieve the $\Gamma _i\sim \theta$ and $\Gamma _{\mathrm{r}}\sim \theta$ relations by fitting the FEM-simulated spectra obtained at different *θ* with the CMT expression Eq. (2) (see Fig. S5 for more details). The retrieved $\Gamma _i\sim \theta$ and $\Gamma _{\mathrm{r}}\sim \theta$ relations are depicted in Fig. 2d, which well explained the obtained line shapes. We note that the absorptive damping rate is quite insensitive to the incident angle (Γ_i_(*θ*) = Γ_*i*_(0)), which is reasonable since this parameter is mainly determined by the constituent materials. Meanwhile, $\Gamma _{\mathrm{r}}$ also exhibits a weak dependence on *θ*, which explains why (nearly) perfect absorption can occur at all incident angles since the perfect-absorption condition $\Gamma _i(\theta ) = \Gamma _r$ (i.e., the critical damping condition^45^) can be approximately satisfied at all off-normal incident angles as long as the condition is met at the normal incident angle.

We use a simple model to explain why $\Gamma _{\mathrm{r}}$ exhibits a very weak dependence on *θ* in such a case. As discussed in ref. ^46,47^, in the lowest order approximation, $\Gamma _r(\theta )$ should be proportional to the radiation power of a single constituent meta-atom being excited in the direction specified by *θ*. Here, the MIM meta-atom supports a magnetic mode with $\vec m$ polarized along the $\hat y$ direction, which, after being excited, radiates nearly equally in all directions within the *x–z* plane (see Fig. S6 for the simulated radiation pattern) in the lowest order approximation, which explains why $\Gamma _{\mathrm{r}}$ is nearly independent of *θ* in this case.

The above analysis clearly reveals the origins of the angular dispersion in a metasurface—the inter-meta-atom couplings that dictate $f_r(\theta )$ and the radiation property of a single meta-atom that dictates $\Gamma _r(\theta )$. These physical understandings provide us with two different approaches to control the angular dispersions of metasurfaces, as we explain in the following subsections.

### Approaches to control the angular dispersions of metasurfaces

#### Manipulating $f_r(\theta )$: incident-angle-insensitive meta-absorbers

We first illustrate how to control the angular dispersion of a metasurface by manipulating its $f_r(\theta )$ relation. According to Eq. (1), we understand that the $f_r(\theta )$ relation of a given metasurface can be efficiently controlled by *designing* the plasmonic couplings among adjacent meta-atoms. In the following, we take an incident-angle-insensitive meta-absorber as an example to illustrate how the idea works.

**Fig. 3:**
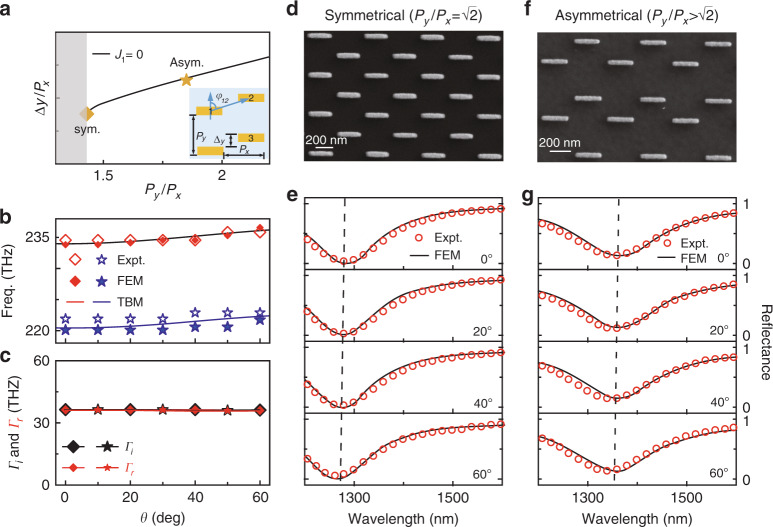
Experimental demonstrations of two incident-angle-insensitive meta-absorbers. **a** $\Delta y/P_x$ as a function of $P_y/P_x$ obtained by numerically solving Eq. (3). The inset depicts the configuration of the metasurfaces under consideration. **b** $f_r\sim \theta$ relations, obtained from experiments, FEM simulations and TBM calculations, for meta-absorbers with symmetrical and asymmetrical configurations. **c** $\Gamma _i\sim \theta$ and $\Gamma _r\sim \theta$ relations, retrieved from FEM simulations, for meta-absorbers with symmetrical and asymmetrical configurations. **d**, **f** SEM images and **e**, **g** measured and simulated reflectance spectra of the fabricated perfect meta-absorbers with symmetrical and asymmetrical configurations under excitation of TM-polarized light at different incident angles. The geometrical parameters of the meta-absorber with the symmetrical configuration are *P*_*x*_ = 270 nm, *P*_*y*_ = 400 nm, *L*_*x*_ = 240 nm, *L*_*y*_ = 50 nm, Δ*y* = 200 nm, *t* = 30 nm, *t*_*s*_ = 45 nm and *t*_*m*_ = 150 nm. For the device with the asymmetrical configuration, *P*_*y*_ = 500 nm and Δ*y* = 190 nm, with the other parameters remaining the same as in the symmetrical configuration

Eq. (1) suggests that $J_1 = 0$ is the criterion to realize a (nearly) angular-dispersionless meta-device since *J*_1_ is the largest inter-row coupling parameter. As demonstrated in ref. ^40^, *J*_1_ is the sum of all coupling strengths between meta-atoms belonging to adjacent rows. Instead of enlarging the inter-row distance to reduce the coupling *J*_1_, here, we propose to achieve this goal by *rearranging* the inter-meta-atom configurations of a metasurface. Specifically, considering a generic lattice configuration (see the inset in Fig. 3a) with each row shifted a distance $\Delta y$ along the *y* direction with respect to its adjacent row and then considering only the lower-order inter-meta-atom couplings, we find that the condition to realize an angular-dispersionless meta-absorber is

3 $$J_1 \approx 2\left( {t_{12} + t_{13}} \right) = 0$$

with $i = 1,2,3$ labeling the meta-atoms located at lattice points belonging to two adjacent rows (see the inset in Fig. 3a). Since all *t*_*ij*_ parameters can be quantitatively computed using our photonic TBM, we can solve Eq. (3) to obtain a series of solutions of $\Delta y$ under different values of $(P_x,P_y)$ and depict the results in Fig. 3a as a black solid line (see Fig. S8 in Supplementary Information for more details).

We now choose two particular solutions from Fig. 3a to experimentally demonstrate our predictions. The first one is a special solution (with $P_y/P_x = \sqrt 2$) corresponding to a lattice configuration with a symmetrical shift (marked by a square in Fig. 3a), while the second one (with $P_y/P_x\, > \,\sqrt 2$) does not exhibit such symmetry (marked by a star in Fig. 3a). We fabricate two meta-devices according to these two solutions (see Fig. 3d, f for their SEM images) and then experimentally characterize their reflectance spectra under different incident angles. The experimental results are depicted in Fig. 3e, g, which are in good agreement with the corresponding FEM simulations (see Fig. S9 for more measured and simulated results). Most importantly, both experiments and simulations unambiguously demonstrate that the two meta-devices can perfectly absorb light at two fixed wavelengths (1275 and 1353 nm), *insensitive* to variations in the incident angle.

To further understand these results, we employ the same theoretical tools as in the last subsection to analyze both the $f_r(\theta )$ and $\Gamma _r(\theta )$ relations of the two fabricated devices. The $f_r(\theta )$ curves of the two meta-devices, calculated with the photonic TBM for realistic structures without any fitting parameters, are compared with both experimental and simulation results in Fig. 3b. Clearly, the resonance frequencies of the two carefully designed meta-devices exhibit much weaker angle dependences than that of the “non-designed” meta-absorber studied in the last subsection. We next retrieve the two damping parameters ($\Gamma _i$ and $\Gamma _r$) from the FEM-simulated reflection coefficients at different incident angles and depict their *θ* dependence in Fig. 3c. Again, both $\Gamma _i$ (black symbols) and $\Gamma _r$ (red symbols) are quite *θ* insensitive, as expected, sharing the same physics as those discussed in the Section “Revealing the origins of angular dispersions in metasurfaces”, also explaining why perfect absorption can occur at all incident angles.

We now provide a simple picture to understand the above two solutions. According to the effective model for photonic coupling established in ref. ^42^, we understand that *t*_12_ and *t*_13_ are proportional to the dipolar interactions between the two magnetic dipoles possessed by two MIM meta-atoms and can be analytically written as

4 $${t}_{1i}{\mathrm{ = }}\frac{{\omega _0\mu _0}}{{8\pi \left\langle {\Phi {\mathrm{|}}\Phi } \right\rangle }}\frac{{\left( {1{\mathrm{ - }}3\cos ^2\varphi _{1i}} \right)\left| {\vec m} \right|^2}}{{\left| {\vec r_{1i}} \right|^3}}\,\left( {i = 2\,{\rm{or}}\,3} \right)$$

where $\left\langle {\Phi {\mathrm{|}}\Phi } \right\rangle$ is the normalized energy stored in a single meta-atom, $\vec r_{1{\mathrm{i}}}$ is the vector linking the centers of the two meta-atoms, and $\varphi _{12} = {\mathrm{cos}}^{ - 1}\left( {\Delta y/\sqrt {P_x^2 + \Delta y^2} } \right)$ and $\varphi _{13} = \cos ^{ - 1}\left( {(P_y - \Delta y)/\sqrt {P_x^2 + (P_y - \Delta y)^2} } \right)$ are the angles between $\vec r_{1{\mathrm{i}}}$ and ${\vec{\mathrm m}}$. Inputting this information into Eq. (3), we can thus solve Eq. (3) to obtain the solutions of $\Delta y$ under different values of $(P_x,P_y)$. Moreover, such analytical solutions uncover the nature of these two solutions, which cannot be easily understood based only on numerical calculations. Obviously, *t*_12_ and *t*_13_ are functions of *φ*_12_ and *φ*_13_, respectively, which are in turn functions of $\Delta y$. In the case of $P_y/P_x\, < \,\sqrt 2$ (the shaded region in Fig. 3a), $t_{12} + t_{13}$ are always positive regardless of how one varies $\Delta y$, and thus, no solution exists for Eq. (3). Meanwhile, in the special case of $P_y/P_x = \sqrt 2$, we find a particular solution $\Delta y = P_y/2$ of Eq. (3), which essentially makes $t_{12} = t_{1{\mathrm{3}}} = 0$. In fact, such a “magic” angle that makes $t_{12} = 0$ has also been discovered in previous studies^40^. Finally, under the condition of $P_y/P_x\, > \,\sqrt 2$, we find that *t*_12_ and *t*_13_ must exhibit *opposite* signs, and by varying $\Delta y$, one can always find a solution to make $t_{12} = - t_{13}$ and thus satisfy Eq. (3) (see Fig. S8 in Supplementary Information for more details).

Before concluding this section, we emphasize that our approach provides an alternative yet efficient way to control the angular dispersion of a metasurface without changing its periodicity or constituent meta-atoms, distinct from previous attempts that typically rely on enlarging the inter-meta-atom separations^48,49^.

#### Manipulating $\Gamma _r(\theta )$: incident-angle-selective meta-absorber

**Fig. 4:**
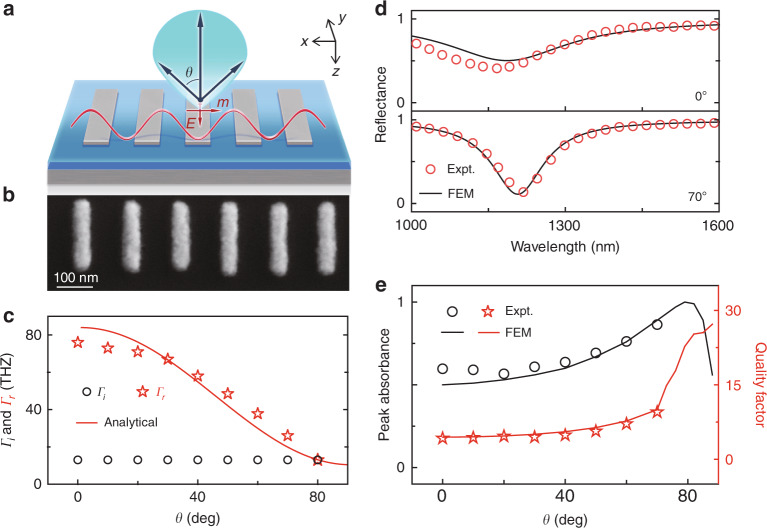
Experimental demonstration of an incident-angle-selective meta-absorber. **a** Schematic of the radiation pattern of an MIM meta-atom excited by TE-polarized light. **b** SEM image of the fabricated sample. **c** $\Gamma _i(\theta )$ and $\Gamma _r(\theta )$ relations of the metasurface retrieved from FEM simulations, compared with the $\Gamma _r(\theta )$ relation calculated by the analytical expression Eq. (5). **d** Measured and simulated reflectance spectra of the sample illuminated by TE-polarized light at different incident angles. **e** Measured and simulated peak absorbance and quality factor of the resonance for the metasurface illuminated by TE-polarized light at different incident angles. Here, silver (Ag) is used as a metallic material. The geometrical parameters of the meta-device are *P*_*x*_ = 120 nm, *P*_*y*_ = 300 nm, *L*_*x*_ = 50 nm, *L*_*y*_ = 200 nm, *t* = 30 nm, *t*_*s*_ = 50 nm and *t*_*m*_ = 150 nm

We now identify the role played by $\Gamma _r(\theta )$ in controlling the angular dispersion of a metasurface. As shown in Fig. 4a, we now study an MIM metasurface similar to that in the Section “Revealing the origins of angular dispersions in metasurfaces” but illuminated by obliquely incident light with transverse electric (TE) polarization. We fabricate a sample (see Fig. 4b for its SEM image) and experimentally characterize its reflectance spectra under TE-polarized illumination at different incident angles *θ*. Figure 4d illustrates the measured reflectance spectra as *θ* varies from 0° to 70°, which are in good agreement with the corresponding FEM simulations (see Fig. S10 for more experimental and simulation results). Compared with Fig. 2e, we find that the system now exhibits completely different angular dispersions. Specifically, while the spectrum-dip frequency does not exhibit a dramatic dependence on *θ*, the resonance bandwidth shrinks and the peak absorption gradually enhances as *θ* increases. These features result in significant modulation of the final optical response (e.g., the line shape) of the metasurface under study.

To understand this unusual angular-dispersion behavior, we follow the same strategy as in the Section “Revealing the origins of angular dispersions in metasurfaces” to retrieve the CMT parameters $\Gamma _r$ and $\Gamma _i$ at different incident angles *θ* and then depict the obtained $\Gamma _r(\theta )$ and $\Gamma _i(\theta )$ relations in Fig. 4c. In sharp contrast with those depicted in Figs. 2d and 3c, $\Gamma _r$ now obviously decreases as *θ* increases. As a result, while the MIM metasurface is located in the underdamped regime (i.e., $\Gamma _r\, > \,\Gamma _i$) with very small absorption under normal incidence^45^, as *θ* increases, the system gradually moves to the critical-damping line defined by $\Gamma _r = \Gamma _i$, leading to significantly enhanced absorption (see Fig. 4e). In particular, at a sufficiently large incident angle *θ* = 80°, the critical coupling condition $\Gamma _r = \Gamma _i$ can be satisfied, leading to 100% absorption at this particular incident angle, as verified by our full-wave simulations on the realistic structure^45^. This angle-selective absorption property is more clearly illustrated in Fig. 4e, where the peak absorbance and the quality factor (inversely proportional to $\Gamma _r$) of our meta-device are shown as functions of *θ*, obtained by both experiments and simulations. Obviously, such a fascinating property is dictated by the unusual *θ* dependence of $\Gamma _r$, which is another factor to control the optical response of a metasurface by varying the incident angle.

We now explain why our metasurface can exhibit such an interesting $\Gamma _r(\theta )$ relation. As we discussed in the Section “Revealing the origins of angular dispersions in metasurfaces”, the $\Gamma _r(\theta )$ relation is essentially dictated by the radiation pattern of the meta-atom, which is still a magnetic dipole in the present system. However, under the excitation of TE-polarized light, the relevant radiation pattern of the magnetic dipole is now on the *x-z* plane, in which the magnetic moment direction lies ($\vec m||\hat x$). Therefore, the radiation pattern of the dipole is completely different from that studied in the Section “Revealing the origins of angular dispersions in metasurfaces”. Neglecting the influences of the metallic back plane, we understand that the radiation power of the magnetic dipole should be proportional to $\cos ^2\theta$ due to the transverse nature of the radiated EM waves (see Fig. S7 for the simulated radiation pattern). Indeed, we find that the analytical expression^50^

5 $$\Gamma _r\left( \theta \right) = 10.4 + 73.6 \ast {\rm{cos}}^2\theta$$

can describe the realistic $\Gamma _r(\theta )$ curve of our metasurface very well (see Fig. 4c), which further explains why our meta-device can exhibit such intriguing angle-dependent properties, as shown in Fig. 4e. We note here that the background constant (e.g., 10.4) in Eq. (5) must be contributed by the metallic ground plane, which makes the MIM meta-atom slightly different from a pure magnetic resonator.

We emphasize that our strategy to control angular dispersions is general enough and can be applied to other types of metasurfaces than the MIM ones exhibiting polarization-*locked* angular dispersions (see Figs. 2–4). As an illustration, we numerically examine the angular dispersion of a carefully designed transmissive metasurface composed of a periodic array of U-shaped resonators. We find that both the *f*_*r*_ and $\Gamma _r$ of such a metasurface exhibit strong angular dispersion under a certain excitation polarization (see Fig. S11 in Supplementary Information), different from the MIM metasurfaces studied here. The inherent physics is that the angular dispersion of a metasurface is *not* determined by the polarization but rather by the inter-resonator couplings and single-resonator radiation, which are fully controlled by the local resonating structures.

#### Applications

Based on the physical understandings gained in the last section, we can realize functional meta-devices with angle-dependent functionalities by carefully designing both the constituent meta-atoms and the local environments of the meta-atoms. In this section, we demonstrate two such meta-devices through experiments and simulations.

#### An angle-multiplexed meta-polarizer

**Fig. 5:**
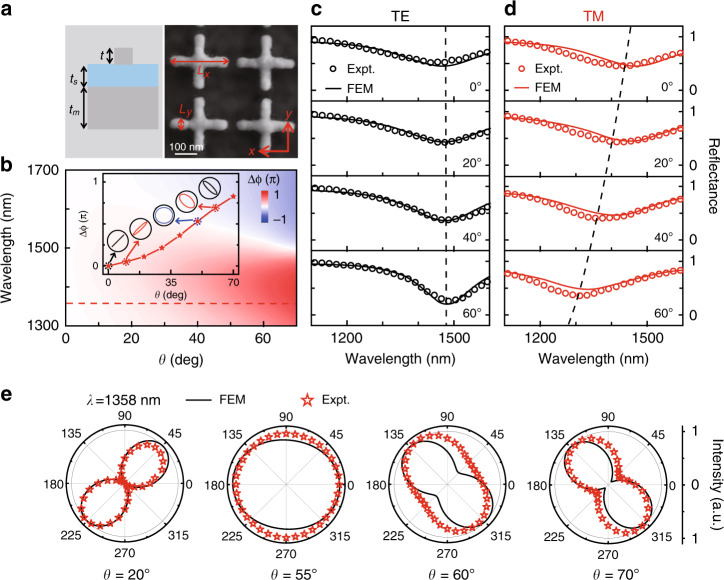
Experimental demonstration of an angle-multiplexed meta-polarizer. **a** Side-view schematic (left) and top-view SEM image (right) of the designed/fabricated metasurface. **b** FEM-simulated reflection-phase difference between TE and TM polarizations versus wavelength and incident angle. Inset: TE–TM reflection phase difference versus incident angle, obtained by FEM simulations at the wavelength of 1358 nm (corresponding to the red dashed line), implying that the reflected beam can exhibit different polarization states. **c**, **d** Measured and simulated reflectance spectra of the fabricated meta-device illuminated by TE- and TM-polarized light at different incident angles. **e** Normalized power patterns of the metasurface-reflected light filtered by a linear polarizer with rotating polarization direction, obtained by experiments and simulations at the wavelength of 1358 nm under different excitation light incident angles. The geometrical parameters of the meta-device are *P*_*x*_ = *P*_*y*_ = 280 nm, *L*_*x*_ = 250 nm, *L*_*y*_ = 50 nm, *t* = 30 nm, *t*_*s*_ = 50 nm and *t*_*m*_ = 150 nm

We first realize an angle-multiplexed meta-polarizer based on a periodic metasurface with carefully designed angular dispersion. As schematically depicted in Fig. 5a, the designed meta-device is a periodic metasurface with a building block of an MIM meta-atom whose top resonator is a Ag cross exhibiting *x–y* symmetry. Under normal incidence, the *x–y* symmetry of the meta-atom ensures that the whole device exhibits identical reflection-phase spectra when illuminated by light polarized along the *x* and *y* directions (see Fig. S12a). As we vary the **k** vector of incident light in the *x–z* plane, the two excitation light components with in-plane **E** vectors pointing along the *x* and *y* directions now correspond to TM- and TE-polarized incident light, respectively. As illustrated in Fig. 5c, d, a metasurface can exhibit distinct angular dispersions for TE and TM excitations. Therefore, we can design the metasurface to purposely enlarge its difference in the optical responses under TE and TM excitations at oblique incident angles so that it can function as a waveplate at a particular off-normal incident angle, although it behaves as an isotropic mirror under normal incidence.

Figure 5c, d depicts how the FEM-simulated reflectance spectra (lines) of the designed sample vary as *θ* increases for the two different polarizations. In the TE case, the reflection-dip frequency barely changes, while the reflection-dip bandwidth obviously shrinks; in the TM case, the resonance frequency undergoes a considerable blueshift with a quite stable reflection-dip bandwidth. All these features are consistent with those presented in the last section and have been well explained by our theory (see Fig. S13 in the Supplementary Information for more details). Such opposite trends significantly enlarge our system’s “*effective optical anisotropy*” at oblique angle incidences, as shown in Fig. 5b (also in Fig. S12b), where the phase difference $\Delta \phi = \phi _{\mathrm{TE}} - \phi _{\mathrm{TM}}$ is depicted as a function of *θ* and the working wavelength *λ*. Such a strong *θ* dependence of $\Delta \phi$ offers a novel possibility to realize an angle-multiplexed polarization controller of reflected light. As shown in the inset in Fig. 5b, at the wavelength of $\lambda = 1358\,{\mathrm{nm}}$, $\Delta \phi$ continuously changes from 0 to 0.8π as *θ* increases from 0° to 70°. Therefore, when illuminating the metasurface by linearly polarized light with a tangential **E** vector lying at an angle 45° with respect to the *x*-axis (which can be decomposed into TE and TM modes with equal amplitudes), the reflected light must take a polarization state that can be continuously modulated by varying *θ* (see the inset in Fig. 5b).

We fabricate a sample (see the right panel in Fig. 5a for its SEM image) and experimentally demonstrate all of the above predictions. We first measure the reflectance spectra of our sample under illumination by TE- and TM-polarized light at different incident angles and depict the results as open circles in Fig. 5c, d (see Fig. S14 for more experimental and simulated results). Excellent agreement is found between the experimental and simulation results. In addition, the *θ* dependences of the *f*_*r*_, $\Gamma _i$, and $\Gamma _r$ of our meta-device for both TE and TM polarizations are in good agreement with our theoretical analyses (see Fig. S15 in Supplementary Information).

We next employ our macroscopic angular resolved spectrometer to characterize the polarization states of light reflected by the meta-device for different incident angles. In our experiments, we illuminate the sample with light beams at different incident angles but with correct polarization angles as described above and then analyze the polarization states of the reflected light beams by measuring the power of the received signals passing through a linear polarizer placed in front of the sample, which is then rotated to cover the full 360° range. Figure 5e illustrates the evolution of the measured polarized field patterns for light beams reflected at different incident angles. Careful analysis reveals that these patterns are consistent with the polarization states predicted in Fig. 5b, supported by the good agreement between the measured patterns (symbols) and those (solid lines) calculated based on the predicted polarization states. Specifically, while the reflected light exhibits a linear polarization under normal incidence (see Fig. S16b), its polarization state gradually changes from an elliptical polarization (*θ* = 20°) to a circular polarization (*θ* = 55°), then to an elliptical polarization again (*θ* = 60°) but with the principle axis rotated by 90°, and finally to an elliptical polarization that is very close to a cross-polarized linear polarization (*θ* = 70°) (see Fig. S16 for more measured/simulated results). Quantitatively, for the case of *θ* = 55°, the measured (simulated) degree of circular polarization $\left( {\mathrm{DOCP} = \left| {a_r} \right|^2/\left( {\left| {a_r} \right|^2 + \left| {a_l} \right|^2} \right)} \right)$ for the reflected light reaches 93.2% (83.7%) with an absolute reflection efficiency of 56.6% (57.5%), where *a*_*r*_ and *a*_*l*_ denote the coefficients of the right and left circularly polarized components of the reflected beam. Meanwhile, for the *θ* = 70° case, the measured (simulated) polarization conversion ratio $\left( {\mathrm{PCR} = a_{cro}^2/\left( {a_{co}^2 + a_{cro}^2} \right)} \right)$ for the reflected light is 79.7% (92.3%) with an absolute reflection efficiency of 68.6% (69%), where *a*_*cro*_ and *a*_*co*_ denote the coefficients of the cross- and co-polarized components (with respect to that of the incident light) of the reflected beam, respectively (see Fig. S17 in Supplementary Information for the evaluation of the absolute working efficiency). The discrepancies between experiments and simulations might be caused by sample imperfections and non-plane wave inputs. We note that the absolute working efficiency and the polarization conversion efficiency of our meta-device can be further improved by using materials with lower losses (see Fig. S18 in Supplementary Information).

#### An angle-multiplexed wavefront controller

The ability to freely control the angular dispersions of metasurfaces also provides us with a new route to realize angle-multiplexed wavefront-control meta-devices based on *inhomogeneous* metasurfaces, as we demonstrate in this subsection. To this end, we need to first find a series of meta-atoms with well-controlled angular dispersions so that their phase responses sensitively depend on the excitation angle. Based on the knowledge gained in the Section “Approaches to control the angular dispersions of metasurfaces”, we understand that such meta-atoms need to contain multiple resonators coupled together so that the inter-resonator couplings can strongly modulate the angular dispersions of the whole meta-atoms.

**Fig. 6:**
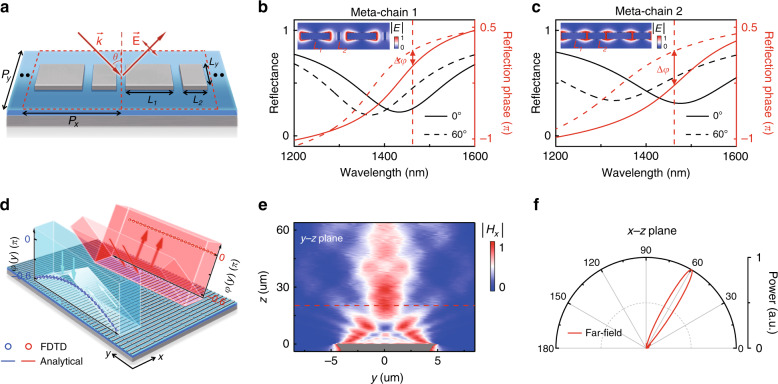
Numerical demonstration of an angle-multiplexed wavefront controller. **a** Schematic of the meta-chain as a build block for the angle-multiplexed bifunctional meta-device, using Ag and SiO_2_ as metallic and dielectric materials. **b**, **c** FDTD-simulated spectra of the reflectance and reflection phase for two different meta-chains (with *L*_1_ = 253 nm, *L*_2_ = 10 nm and *L*_1_ = 192 nm, *L*_2_ = 135 nm) under excitation of TM-polarized light at two different incident angles (0° and 60°). Insets: FDTD-simulated $\left| {\vec E} \right|$ field distributions on the surfaces of the two different meta-chains. **d** Schematic of two distinct functionalities and corresponding reflection-phase distributions of our designed meta-device when illuminated by TM-polarized light at two different incident angles. **e** FDTD-simulated |*H*_*x*_|field distribution on the *y–z* plane for the designed meta-device under normal-incidence excitation with polarization $\vec E||\hat x$ and wavelength $\lambda = 1462\,\,{\mathrm{nm}}$. **f** FDTD-simulated (normalized) far-field scattering pattern on the *x–z* plane for the meta-device illuminated by TM-polarized light at $\theta = 60^ \circ$ and $\lambda = 1462\,\,{\mathrm{nm}}$. Ag is used as a metallic material in this meta-device. The fixed geometrical parameters of the meta-chain are *P*_*x*_ = 350 nm, *P*_*y*_ = 200 nm, *L*_*y*_ = 50 nm, *t* = 30 nm, *t*_*s*_ = 50 nm and *t*_*m*_ = 150 nm

As a proof of concept only, here, we choose to demonstrate a one-dimensional (1D) wavefront controller exhibiting an inhomogeneous phase distribution only along the *y* direction. The meta-atoms forming such a device are a series of 1D chains (e.g., meta-chains), each consisting of two different MIM structures periodically repeated along the *x* direction, as schematically shown in Fig. 6a. By tuning the structural parameters (i.e., *L*_1_ and *L*_2_), we can not only alter a meta-chain’s reflection-phase spectrum under normal incidence but also, more importantly, modulate the difference between the reflection phases measured under normal and off-normal incidences by “tuning” the mutual couplings between two MIM resonators inside the meta-chain. Figure 6b, c depicts the FEM-simulated spectra of the reflectance and reflection phase for two typical meta-chains with different values of *L*_1_ and *L*_2_ (see Fig. S19 and Table S1 in the Supplementary Information for more detailed analyses on systems with different *L*_1_ and *L*_2_). In our calculations, we periodically repeat the meta-chains along the *y* direction to form two infinite metasurfaces whose reflection properties can be unambiguously defined. Due to the weak couplings between adjacent meta-chains (see Fig. S20 in Supplementary Information), the obtained results can well represent the properties of the single meta-chains under study. Choosing the working wavelength of 1462 nm (see the dashed lines in Fig. 6b, c), we find that the two meta-chains indeed exhibit distinct normal-incidence reflection phases as well as distinct angular dispersions manifested by the different values of $\Delta \varphi$ achieved in the two different systems.

Combining different meta-chains to form a metasurface and assuming that each meta-chain exhibits a reflection phase $\varphi ^\theta$ under TM-polarized illumination at incident angle *θ*, we then obtain a meta-device exhibiting a *θ*-dependent phase profile $\varphi ^\theta (y)$. Based on large-scale numerical calculations, we finally distinguish a series of meta-chains (see Table S2 in Supplementary Information for the details of the obtained meta-chains) such that the constructed inhomogeneous meta-device exhibits the following reflection-phase profiles for two incident angles^21^

6 $$\left\{ \begin{array}{l}\varphi _x^{0^ \circ }\left( y \right) = k_0\left( {\sqrt {F^2 + y_{\max }^2} - \sqrt {F^2 + y^2} } \right)\\ \varphi _x^{60^ \circ }\left( y \right) = 0\end{array} \right.$$

where *y*_max_ = 4.6 µm is the half length of the meta-device along the *y* direction and *F* = 20 µm is the focal length. Obviously, Eq. (6) indicates that our meta-device behaves as a focusing lens for normally incident light with polarization $\vec E||\hat x$ but changes to a flat mirror for TM-polarized incident light at $\theta = 60^ \circ$ (see Fig. 6d).

We now numerically verify the above predictions. Assuming that our meta-device is illuminated by normally incident light with polarization $\vec E||\hat x$ at a wavelength of 1462 nm, we employ FDTD simulations to compute the distribution of the scattered field with the incident field extracted. Figure 6e depicts the calculated |*H*_*x*_ | field distribution on the *y–z* plane, showing that the reflected light is indeed focused to a line at a distance 20 µm above the device. We next compute the scattering power pattern of our meta-device when it is illuminated by TM-polarized light at $\theta = 60^ \circ$ with in-plane polarization $\vec E_{||}||\hat x$ (see Fig. 6a for the configuration of the input light) and depict the obtained reflection power pattern on the *x–z* plane in Fig. 6f. That the peak of the reflected signal appears in the specular reflection direction clearly demonstrates the mirror functionality of the device when illuminated by incident light at this angle. Note that our meta-device’s working efficiencies for the lens and mirror functionalities are 7% and 15.3%, respectively. Unfortunately, due to the limitations in our nanofabrication facilities, we were not able to fabricate such a meta-device with the fine characteristic scale. We believe, however, that experimental demonstration of such an idea in different frequency domains would be a very interesting project in the future.

Before concluding this section, we emphasize that our strategy is general enough to realize angle-multiplexed meta-devices with arbitrary intended phase profiles for different incident angles as long as one can design a set of meta-atoms exhibiting incident-angle-dependent phases covering the whole range of 360° at the desired frequencies.

## Discussion

In summary, we have combined theory and experiments to reveal that the angular dispersions of metasurfaces are governed by the couplings between meta-atoms and the radiation properties of constituent single meta-atoms. By carefully controlling these two factors, we designed and experimentally realized a series of optical meta-devices with desired angular dispersions, including two incident-angle-insensitive perfect absorbers, one incident-angle-selective perfect absorber, and one angle-dependent multifunctional meta-polarizer. Finally, we numerically demonstrated an angle-multiplexed wavefront-control meta-device employing meta-atom arrays with predetermined angular dispersions. Our findings pave the way to realizing angle-dependent multifunctional meta-devices, which significantly expand the wave-control capabilities of metasurfaces and may stimulate the realization of multifunctional meta-devices for versatile applications.

## Materials and methods

### Simulations

We employed FEM simulations using the commercial software COMSOL Multiphysics to study all periodic metasurfaces in this work. For inhomogeneous metasurfaces, we used the numerical software Concerto 7.0 to perform FDTD simulations. The permittivities of Au and Ag were described by the Drude model $$\varepsilon _r\left( \omega \right) = \varepsilon _\infty - \frac{{\omega ^2_p}}{{\omega \left( {\omega + i\gamma } \right)}}$$, with $$\varepsilon _\infty = 9,$$$$\omega _p = 1.37 \times 10^{16}\,{\mathrm{s}}^{ - 1}{\mathrm{,}}$$ and $$\gamma = 12.24 \times 10^{13}\,{\rm{s}}^{ - 1}$$ for Au and $$\varepsilon _\infty = 5,$$ $$\omega _p = 1.37 \times 10^{16}\,{\mathrm{s}}^{ - 1}{\mathrm{,}}$$ and $$\gamma = 4.08 \times 10^{13}\,{\rm{s}}^{ - 1}$$ for Ag, obtained by fitting our experimental results. The SiO_2_ spacer was considered a lossless dielectric with permittivity $$\varepsilon = 2.1$$. Additional losses caused by surface roughness and grain boundary effects in thin films as well as dielectric losses were effectively considered in the fitting parameter *γ*.

### Fabrication

All our meta-devices were fabricated following standard electron-beam lithography (EBL) and lift-off processes. In fabricating these samples, we first used magnetron sputtering to deposit 3 nm Cr, 150 nm Au/Ag, 3 nm Cr and SiO_2_ film on a silicon substrate. Then, the positive resist MMA EL6 (200 nm) and PMMA A2 (80 nm) were spin coated on the substrate coated with Au/Ag and SiO_2_ layers. The meta-atom arrays were next defined using EBL (JEOL 6300) with an acceleration voltage of 100 kV. After exposure, the resist was developed for 30 s in a 3:1 mixture of isopropanol (IPA) and methyl isobutyl ketone (MIBK). Then, 3 nm Cr and 30 nm Au/Ag were subsequently deposited using electron-beam evaporation. Finally, the sample was dipped in acetone for 30 min to perform lift off. The fabricated metasurfaces were imaged using scanning electron microscopy (Zeiss Sigma) to measure the actual dimensions of the nanostructures. All samples had dimensions of 600 µm × 300 µm.

### Optical characterizations

We used a homemade macroscopic angular resolution spectrometer equipped with a broadband supercontinuum white light source, polarizers, a beam splitter, a CCD, and a fiber-coupled grating spectrometer (Ideaoptics NIR2500) to characterize the angular dispersions of our fabricated metasurfaces. The diameter and divergence angle of the incident light were minimized to 130 µm and ~1°, respectively. The sample was placed on a manual rotation stage, which could be rotated to change the incident angle. A fiber-coupled receiver was placed on a motorized rotation stage to detect the signal reflected in the right direction, and the minimum detectable reflection angle was 7°. To measure the reflectance under normal incidence, we used a beam splitter to deflect the normal reflection beam.

## Supplementary information

Supplementary Information
