# Supplementary material for: Controlling angular dispersions in optical metasurfaces
Source: Light Sci Appl. 2020 May 6;9:76. doi: 10.1038/s41377-020-0313-0 (PMC7200735; doi:10.1038/s41377-020-0313-0)
Supplement: Supplementary file 1 — Supplementary Information [file 41377_2020_313_MOESM1_ESM.docx]

**Supplementary Information:**

**Controlling Angular Dispersions in Optical Metasurfaces**

Xiyue Zhang^1$^, Qi Li^1$^, Feifei Liu^1$^, Meng Qiu ^1^, Shulin Sun^2,3^, Qiong He^1,3,4*^,

and Lei Zhou^1,3,4*^

^1^. State Key Laboratory of Surface Physics, Key Laboratory of Micro and Nano Photonic Structures (Ministry of Education), and Department of Physics, Fudan University,
Shanghai 200438, China

^2^.  Shanghai Engineering Research Center of Ultra-Precision Optical Manufacturing, Green Photonics and Department of Optical Science and Engineering, Fudan University,
Shanghai 200433, China

3. Academy for Engineering and Technology, Fudan University, Shanghai 200433, China

4. Collaborative Innovation Center of Advanced Microstructures, Nanjing 210093, China

$ The authors contributed equally to this work

*Corresponding authors: phzhou@fudan.edu.cn; qionghe@fudan.edu.cn

**Section 1. Experimental setup of home-made macroscopic angular resolved spectrometer**


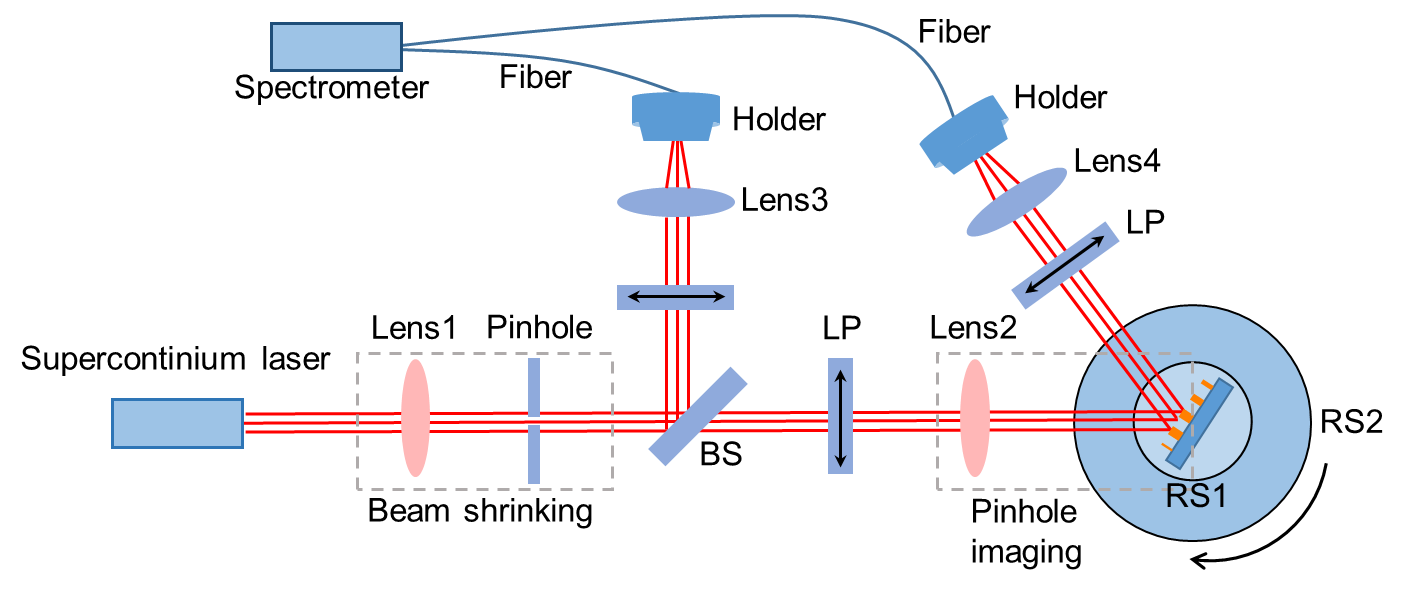


Figure S1. Schematic of home-made macroscopic angular-resolved spectrometer. LP: Linear Polarizer, BS: Beam Splitter, RS: Rotation Stage. Lens1 (Lens 2) is a long (short) focus lens.

**Section 2. Additional experimental and simulated results for Fig. 2** **in the main text**


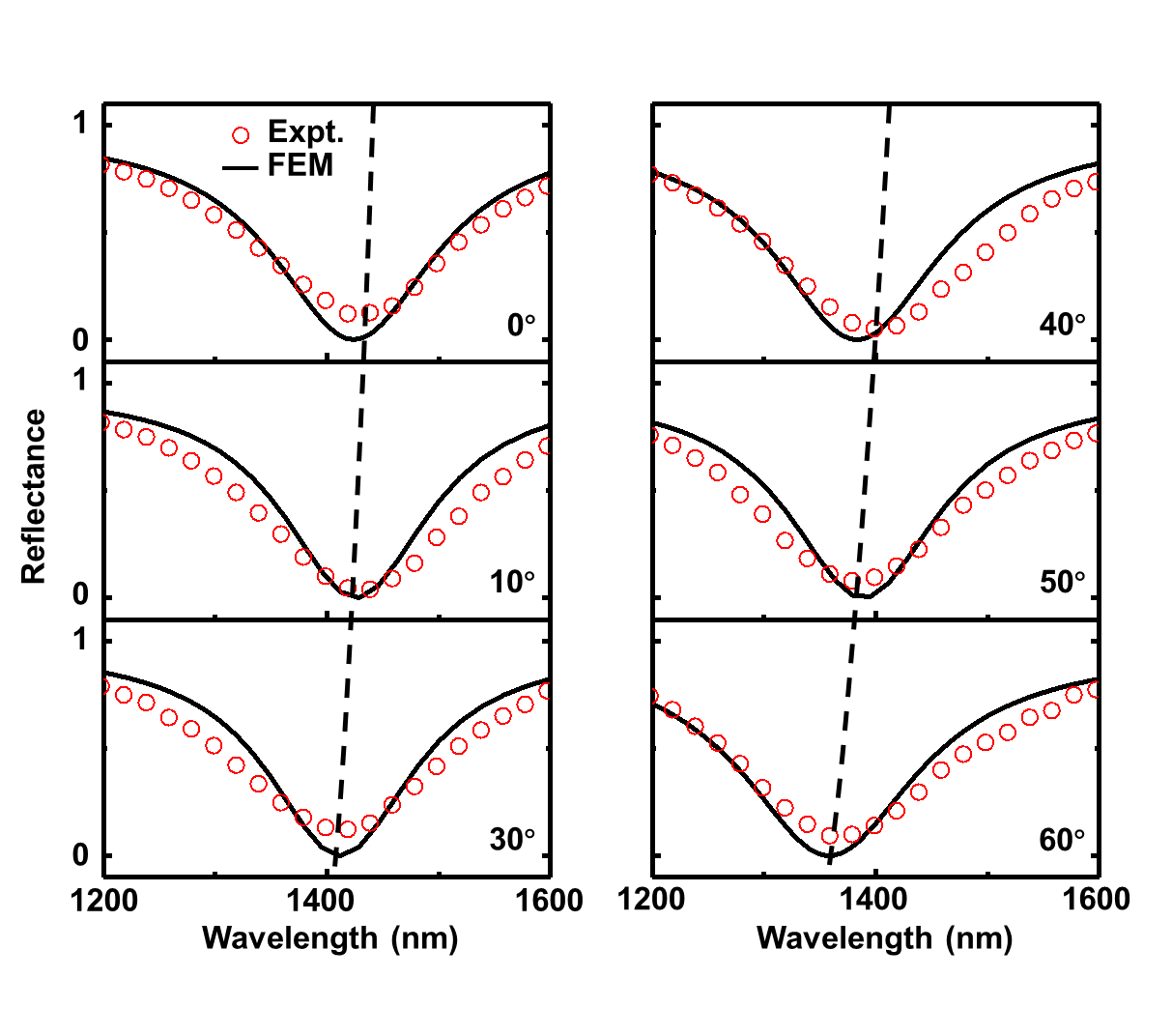


Figure S2. Additional measured/simulated reflectance spectra of the sample studied in Fig. 2 of the main text, shined by TM polarized light at different incident angles.

**Section 3. Details on calculations of the coupling strengths and retrieval of coupled-mode-theory parameters**


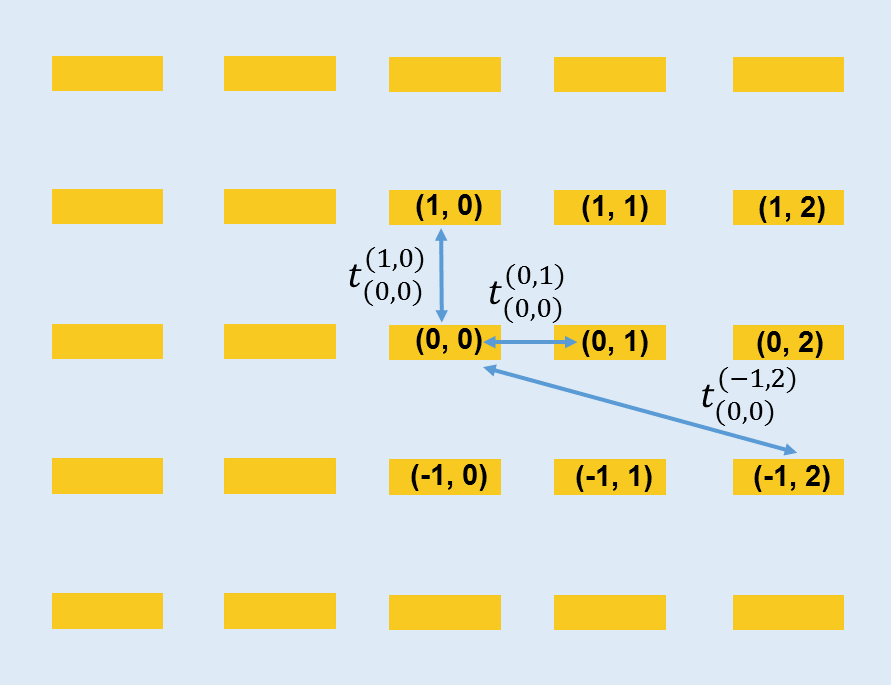


Figure S3. Configuration of part of a periodic metasurface containing
 $5\times5$ meta-atoms.

According to Ref. [1], $J_{0}$, $J_{1}$ and $J_{2}$ are related to inter-meta-atom coupling strengths via the following expressions:

$J_{0}=2\sum_{i} t_{(0,0)}^{(i,0)},\text{ }J_{1}=2\sum_{i} t_{(0,0)}^{(i,1)},\text{ }J_{2}=2\sum_{i} t_{(0,0)}^{(i,2)}$ (1)

Here $\text{t}_{\left\{\text{m},\text{n}\right\}}^{\left\{\text{k},\text{l}\right\}}$is the coupling strength between two meta-atoms located at two different positions labeled by $\left\{m,n\right\}$ and $\left\{k,l\right\}$, respectively. To calculate $J_{1}$, $J_{2}$ and $J_{3}$, we consider the couplings between the central meta-atom and other meta-atoms in a $5\times5$ square ($i\leq2$) as shown in Fig. S3.

The coupling strength between any two resonators can be calculated by the following formula,

$\text{t}_{\left\{\text{m},\text{n}\right\}}^{\left\{\text{k},\text{l}\right\}}\text{= -f}_{0}\frac{\int\overrightarrow{P}_{\left\{\text{m},\text{n}\right\}}^{*}\left(\overrightarrow{\text{r}}\right)*\overrightarrow{E}_{\left\{\text{k},\text{l}\right\}}\left(\overrightarrow{\text{r}}\right)\text{d}\tau}{\left\langle\Phi_{\text{s}}\text{|}\Phi_{\text{s}}\right\rangle}\text{ }(m,n,k,l=0,\pm1,\pm2...)$ (2)

where $\overrightarrow{P}_{\left\{\text{m},\text{n}\right\}}(\vec{r})$ denotes the polarization field inside the meta-atom located at the lattice site $R_{\left\{\text{m},\text{n}\right\}}$, while $\overrightarrow{E}_{\left\{k,l\right\}}(\vec{r})$ denotes the **E**-field distribution generated by the meta-atom located at the lattice site $R_{\left\{\text{k,l}\right\}}$, and $\langle\Phi_{s}|\Phi_{s}\rangle$ is the total EM-field energy stored in a single meta-atom^2^. Since the system under study is essentially an open system, we cannot integrate all the EM energy associated with a single resonator in the whole space, because that will lead to inclusion of the far-field radiations which do not belong to the near-field energy stored inside a resonator. In fact, if we do not extract the energy carried by the far-field radiations from the integration, $\langle\Phi_{s}|\Phi_{s}\rangle$will diverge leading to unphysical results. Figure S4a describes our procedures to calculate $\langle\Phi_{s}|\Phi_{s}\rangle$. We first calculate the total EM energies stored inside spherical spaces with different radius R, and then depict how the obtained energy varies against R (see Fig. S4b). The correct EM-field energy $\langle\Phi_{s}|\Phi_{s}\rangle$ is the intercept of the tangent line of the E-R cure on the *y*-axis, in which the energy carried by the far-field radiations is automatically extracted.


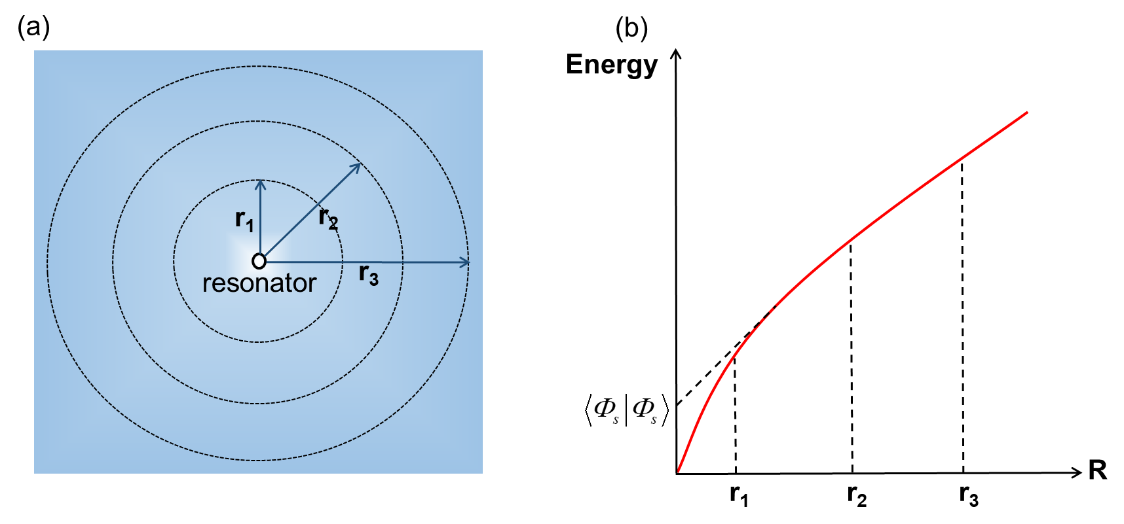


Figure S4. (a) Procedures to calculate the total EM-field energy stored in a single resonator. (b) Schematics of total EM energies stored inside spherical spaces versus different radius R.


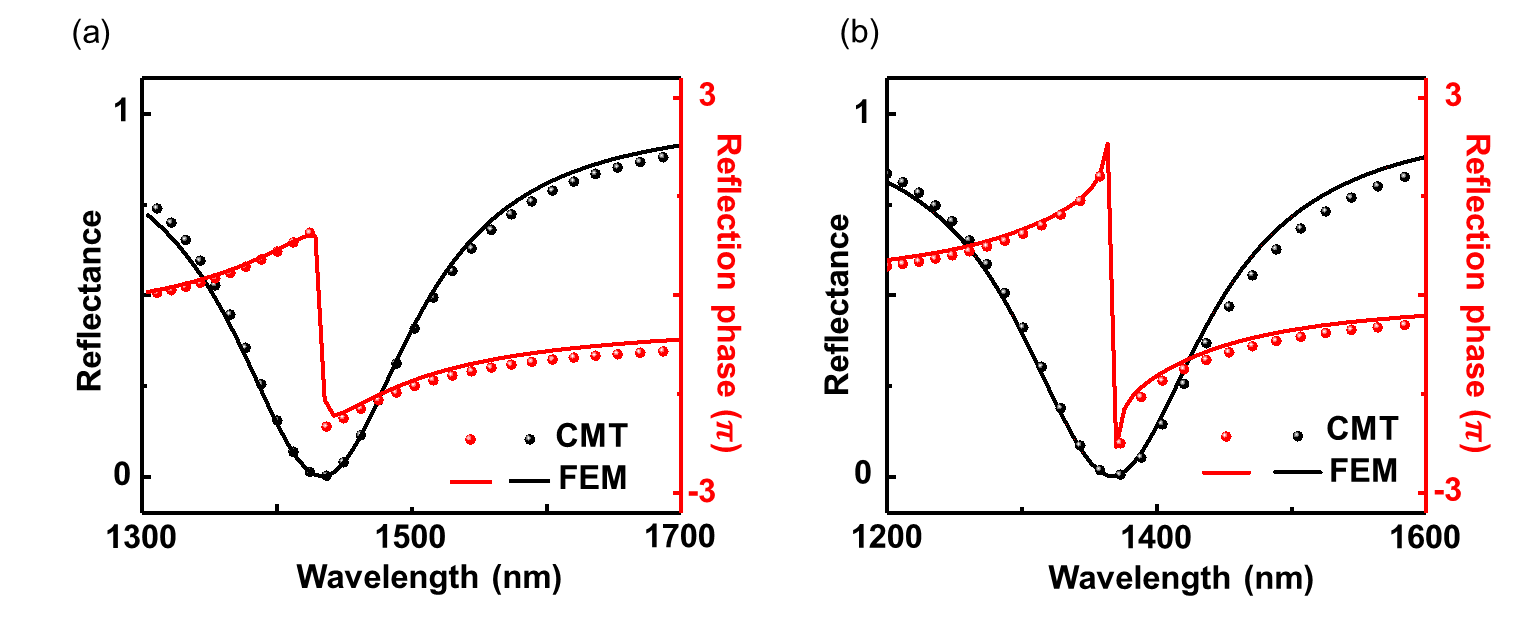


Figure S5. Spectra of reflection amplitude/phase of the metasurface studied in Fig. 2 of the main text, obtained by FEM simulations and CMT calculations at two incident angles: (a) $\theta=0^{\circ}$ and (b) $\theta=60^{\circ}$. Here the damping parameters in the CMT model are $\Gamma_{i}=\Gamma_{r}=\text{36 THz}$ for the case of (a) and$\Gamma_{i}=\text{36.4 THz},\text{ }\Gamma_{r}=\text{36.3 THz}$ for the case of (b).

Figure S5 compares the reflection spectra calculated by the CMT with model parameters depicted in Fig. 2d in the main text and by FEM simulations on the sample studied in Fig. 2 in the main text, at two typical incident angles. Excellent agreement is noted between CMT and FEM results.

**Section 4. FEM-simulated far-field radiation patterns** **of two different MIM meta-atoms on the *x-z* plane**


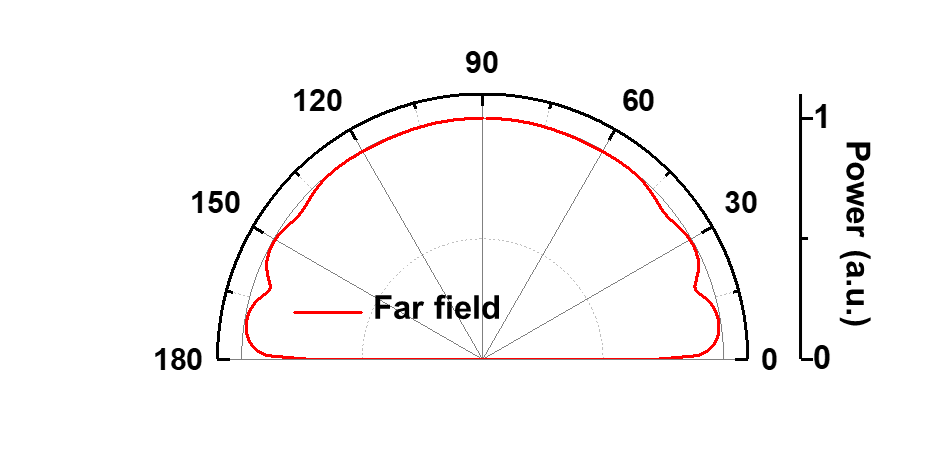


Figure S6. Normalized far-field radiation pattern on the *x-z* plane for a single meta-atom of the metasurface studied in Fig. 2 of the main text, obtained by FEM-simulations at the resonant wavelength of 1424 nm.

**
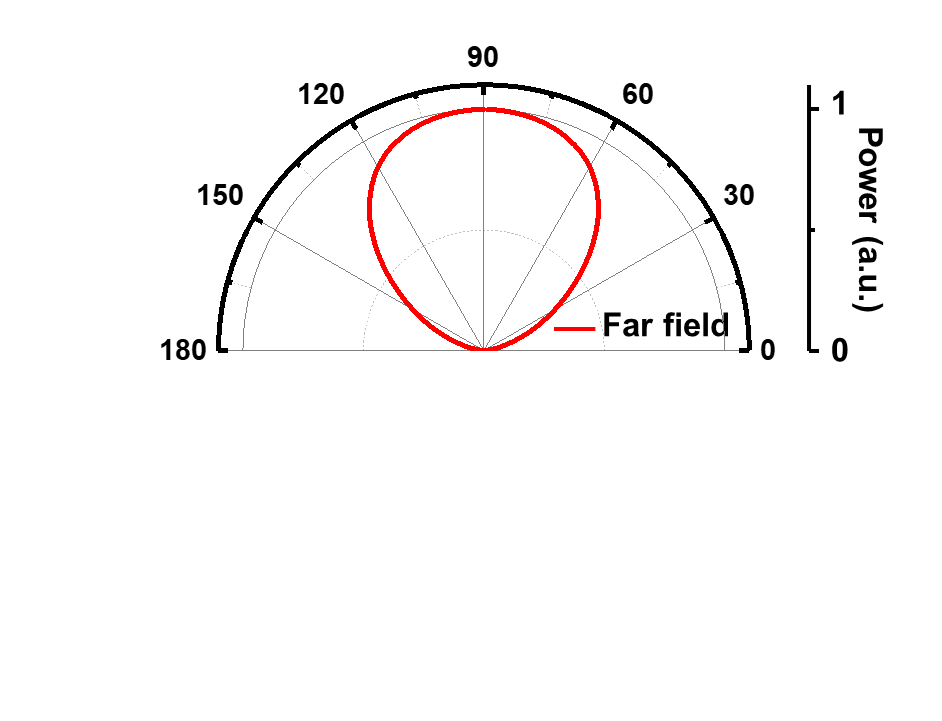
**

Figure S7. Normalized far-field radiation pattern on the *x-z* plane for a single meta-atom of the metasurface studied in Fig. 4 of the main text, obtained by FEM-simulations at the resonant wavelength of 1186 nm.

**Section 5. Additional analytical and experimental/simulation data for Fig. 3 in the main text**

**
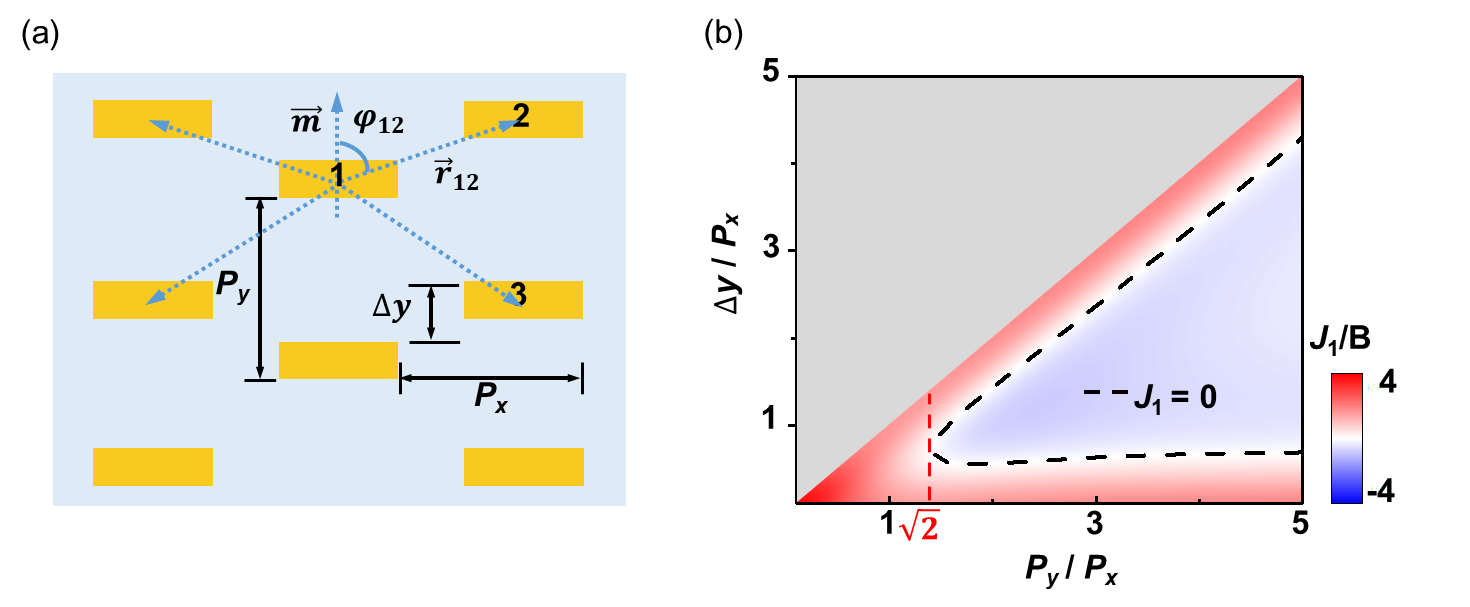
**

Figure S8. (a) Configuration of the super cell structure of the MIM absorber. (b) Analytically-calculated $J_{1}/B$ versus $P_{y}/P_{x}$ and $\Delta y/P_{x}$.

According to Ref. [3], the coupling strength between any two MIM meta-atoms can be simplified as

(3)

where $\vec{m}$ is the magnetic dipole moment, $\vec{r}_{ij}$ is the vector linking the centers of two resonators labeled by *i* and *j*, is the angle between the vector $\vec{r}_{ij}$ and magnetic moment $\vec{m}$, and $\langle\Phi|\left.\Phi\right\rangle$ is a normalized constant which is the EM energy stored in a single meta-atom (see Sec. 3 ). Since $\text{t}_{\text{ij}}$ decays rapidly as $\left|\vec{r}_{ij}\right|$increases, we only need to consider the couplings between two nearest-neighbor meta-atoms pairs (i.e., (1,2) and (1,3) pairs) as we calculate $J_{1}$ (see Fig. S8a). Under this approximation, we find that

(3)

Figure S8b depicts how the analytical calculated $J_{1}/B$ (with $B=\frac{\omega_{0}\mu_{0}\left|\vec{m}\right|^{2}}{4\pi\left\langle\Phi\text{|}\Phi\right\rangle}$) varies as a function of $P_{y}/P_{x}$ and $\Delta y/P_{x}$. The black dashed line denotes the solution for $J_{1}\text{=}0$ (corresponding to the black solid line in Fig. 3a in the main text）.

In case of $P_{y}/P_{x}<\sqrt{2}$,$J_{1}$ is always of positive value, no matter how one varies $\Delta y$, which means that solutions for $J_{1}=0$ do not exist. The gray area in Fig. S6 (b) indicates that $\Delta y>P_{y}$, which is physically not allowed.

For the critical point $P_{y}/P_{x}=\sqrt{2}$(red dashed line), we find that $\Delta y=\frac{P_{y}}{2}$ leads to (corresponding to the square in Fig.3a), which in turn, leads to $\text{t}_{12}\text{= t}_{13}=0$ and finally $J_{1}=0$.

In the case of $P_{y}/P_{x}>\sqrt{2}$, we can always find two solutions of $\Delta y$ to make $t_{12}=-\text{ }t_{13}$ and thus make $J_{1}=0$ satisfied. However, we find that these two solutions are in fact degenerate, since they satisfy $\Delta y_{1}+\Delta y_{2}\equiv P_{y}$ and thus they correspond to the SAME configuration. As the result, we only draw the upper branch of the two solutions in Fig. 3a of the main text.

Figure S9 contains additional experimental/simulation data for the two samples studied in Fig. 3 of the main text, from which we can easily see that the perfect absorption frequencies do not change in both cases.


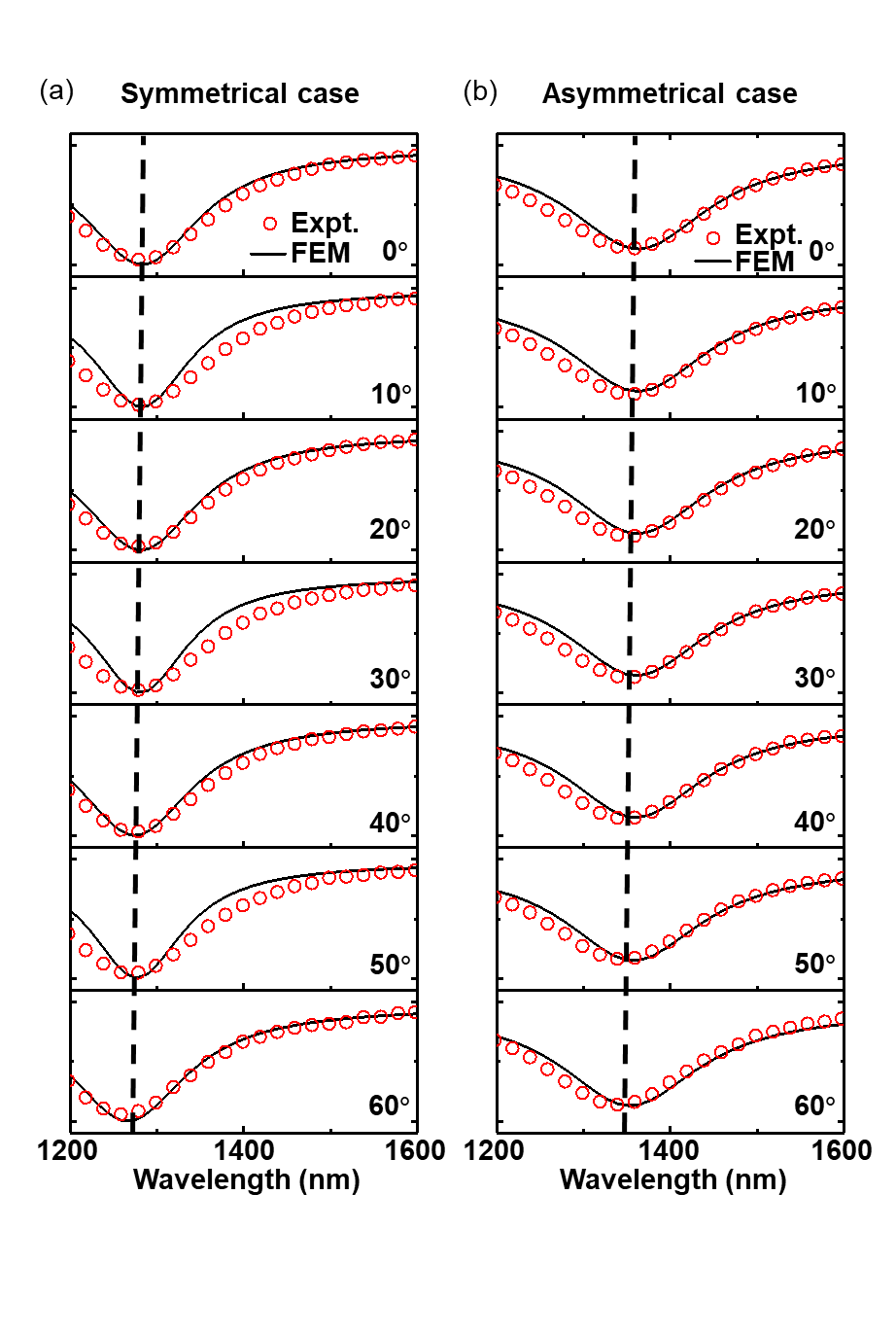


Figure S9. Additional experimental/simulation data for the two samples studied in Fig. 3 of the main text, shined by TM polarized light at different incident angles.

**Section 6. Additional experimental and simulation data for Fig. 4 in the main text**


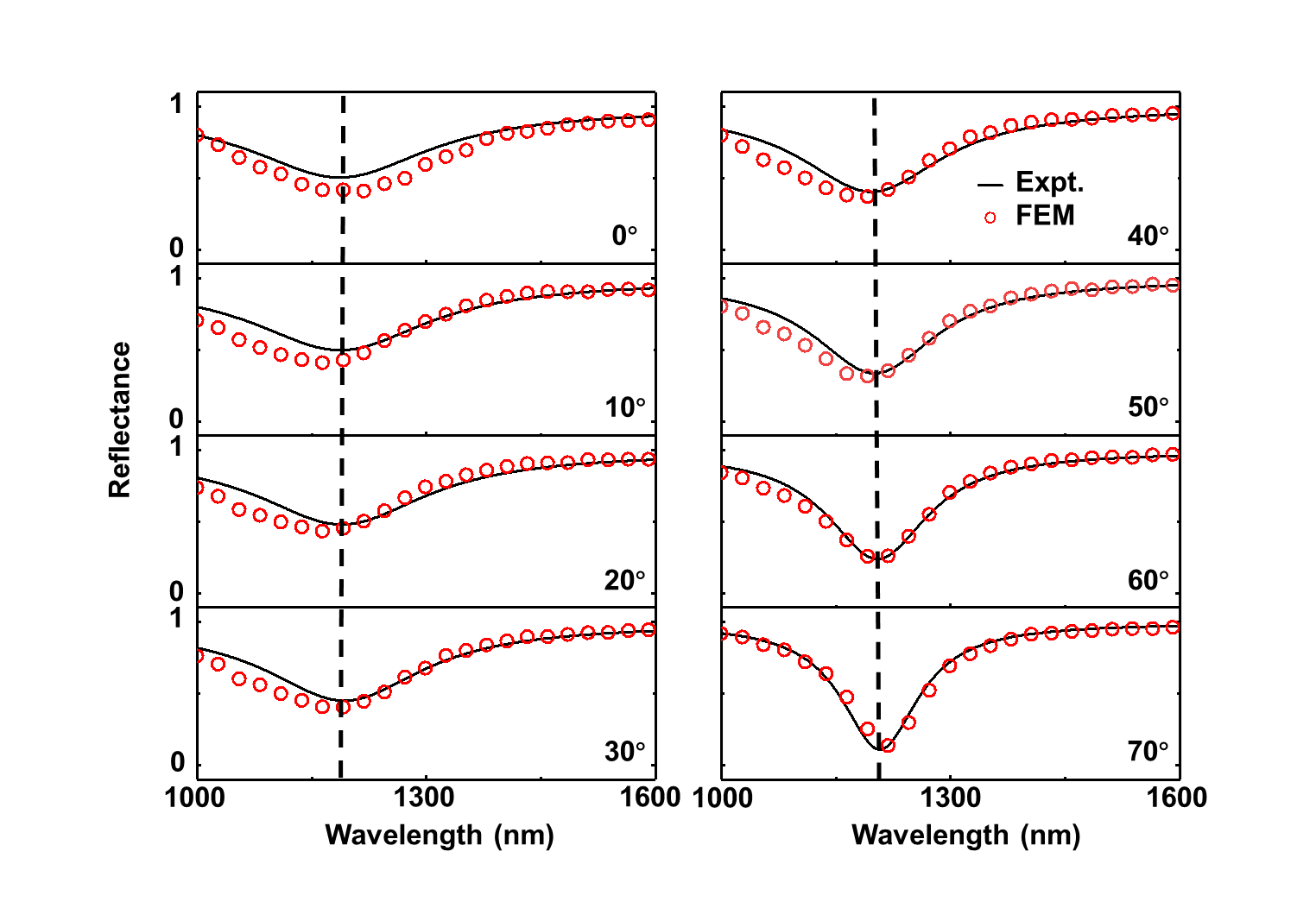


Figure S10. Additional experimental/simulation data for the sample studied in Fig. 4 of the main text, shined by TE polarized light at different incident angles.

**Section 7. Angular dispersions of a transmissive metasurfaces: simultaneous tuning on both $f_{r}(\theta)$ and** $\Gamma_{r}(\theta)$


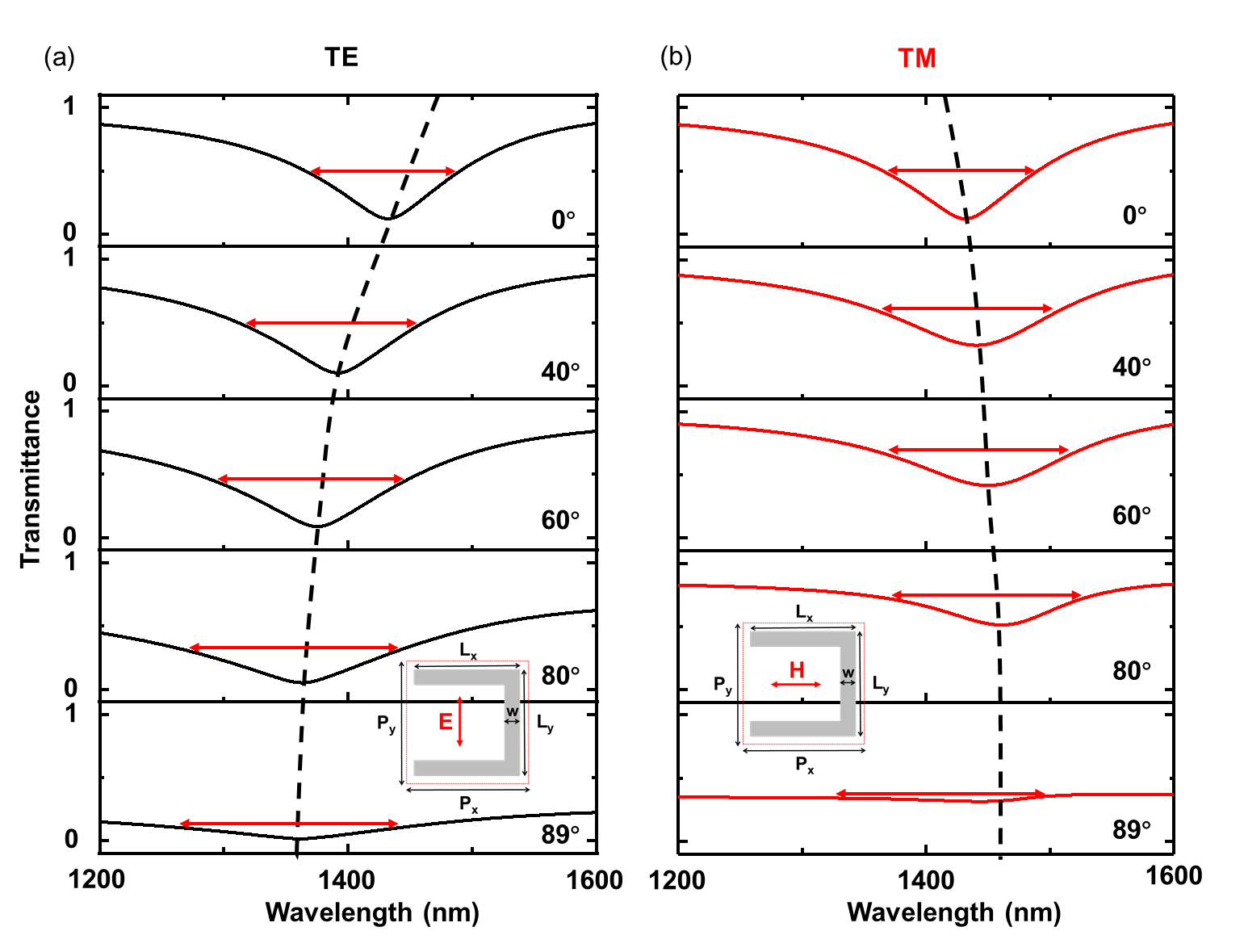


Figure S11. FDTD-simulated transmittance spectra of the transmissive metasurface shined by (a) TE and (b) TM polarized light at different incident angles. Here, the silver U-shaped meta-atom is suspended in the air. Geometrical parameters of the meta-atom are *P_x_* = *P_y_*= 220 nm, *L_x_* = *L_y_* = 200 nm, *w*=30 nm.

We purposely designed a transmissive metasurface composed by array of U-shaped resonators (inset to Fig. S11), and then numerically studied its angular dispersions for different polarizations. Figure S11 shows the FDTD-simulated transmission spectra of the metasurface shined by TE- and TM-polarized lights at different incident angles. Obviously, now both $f_{r}$ and $\Gamma_{r}$can exhibit strongly angle dependences in a certain polarization, which is quite different from the case of MIM systems exhibiting polarization-*locked* angular dispersions. The physics is that now the U-shaped resonator can support both an electric mode and a magnetic mode simultaneously, as shined by a TE or TM polarized light. Therefore, the radiation pattern of such a meta-atom can be very different from a simple resonator, which explains why we observe strong modulation on $\Gamma_{r}$as varying incident angle. Meanwhile, the broken symmetry possessed by such a meta-atom allows us to well control its NFC with adjacent one, thus modulating the resonating frequency $f_{r}$ as varying incident angle. **Section 8. Additional experimental and simulation data for Fig. 5 in the main text**


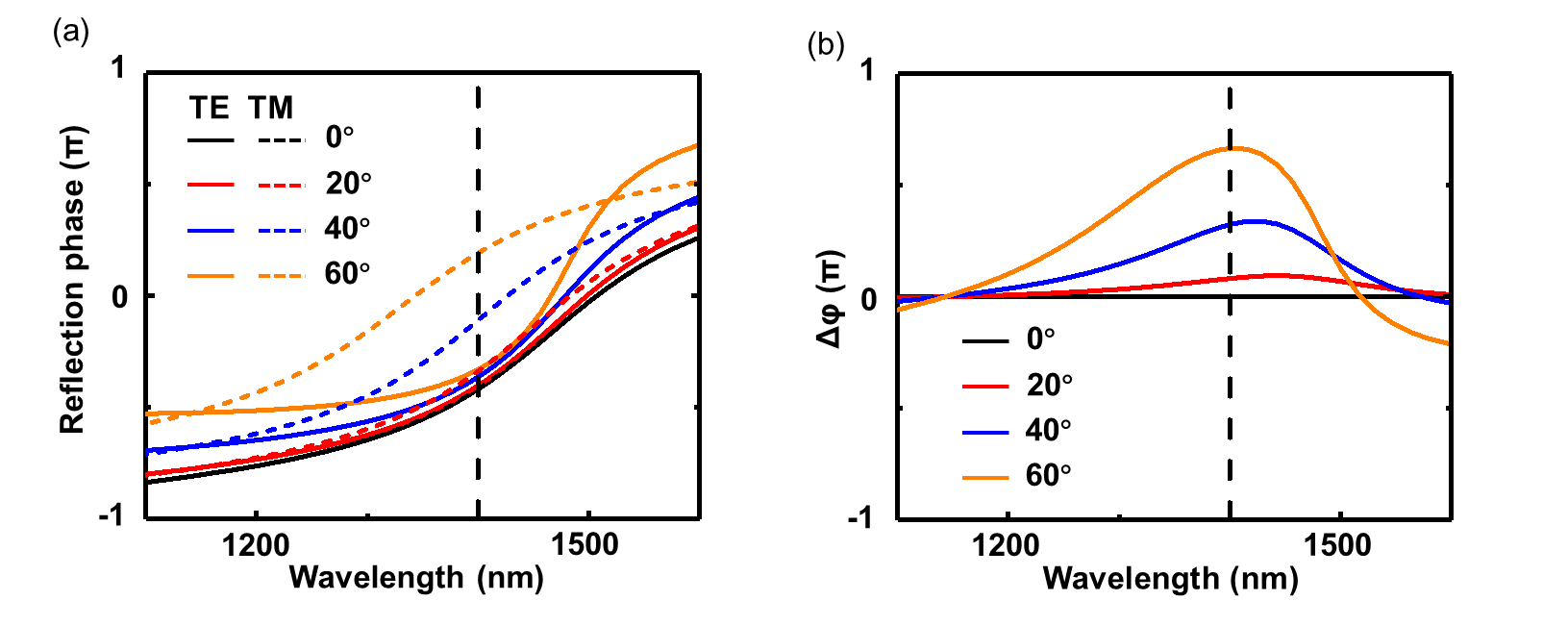


Figure S12. (a) FEM simulated reflection phase spectra for TE- and TM- polarized excitations and (b) reflection-phase difference between TE and TM polarizations for the angle-multiplexed meta-polarizer studied in Fig. 5 of the main text, at different incident angles.


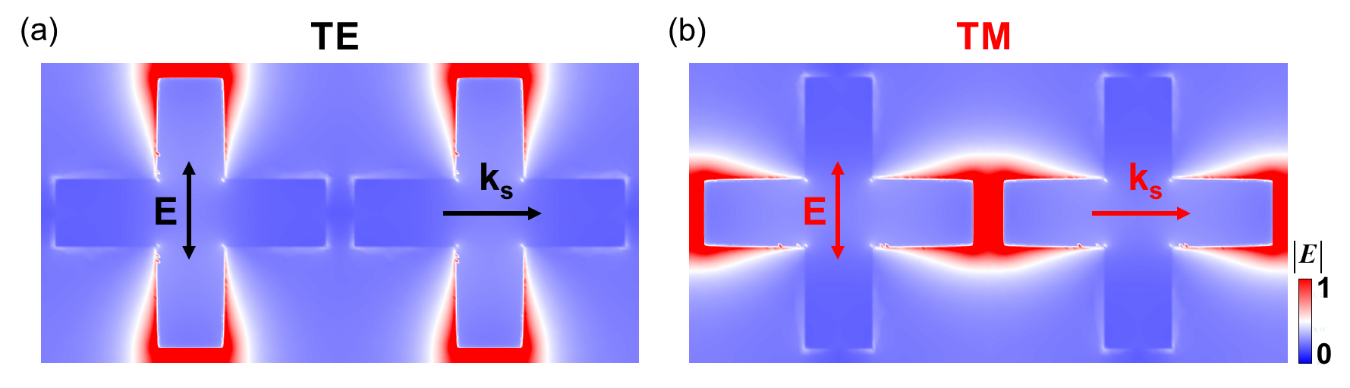


Figure S13. FEM simulated $\left|E\right|$ field distributions on the surface of two adjacent meat-atoms under normal incidence for (a) TE and (b) TM polarization at working wavelength of 1358 nm.

Figure S13 compares the $\left|\vec{E}\right|$ field distributions associated with the excited resonant modes of the designed angle-multiplexed meta-polarizer studied in Fig. 5, shined by normally incident light with two different polarizations. For the TE case, we find that the hot spots in two nearest neighbor meta-atoms are well separated leading to weak near-field coupling (NFC), which explains why the angular dispersion is very weak in such a case (see Fig. 5c in the main text). In constant, the NFC in the TM case must be quite strong since the hot spots on two adjacent meta-atoms are quite close (see Fig. S13b), which is the key reason to account for the strong angular dispersions in this case (see Fig. 5d in the main text). Meanwhile, in the TE (TM) case, the meta-atom supports a magnetic mode with $\vec{m}$ along $\hat{x}$ ($\hat{y}$) direction, which radiates significantly different (nearly equally) to all directions with the *x-z* plane after being excited. Therefore, the significantly different angular dispersions for two polarizations, generated by distinct NFCs and far field radiations, offer us the possibilities to design incident-angle dependent polarization controller.

Figure S14 depicts the measured and FEM-simulated reflectance spectra of the angle-multiplexed meta-polarizer in NIR regime shined by TE- and TM-polarized light at different incident angles.


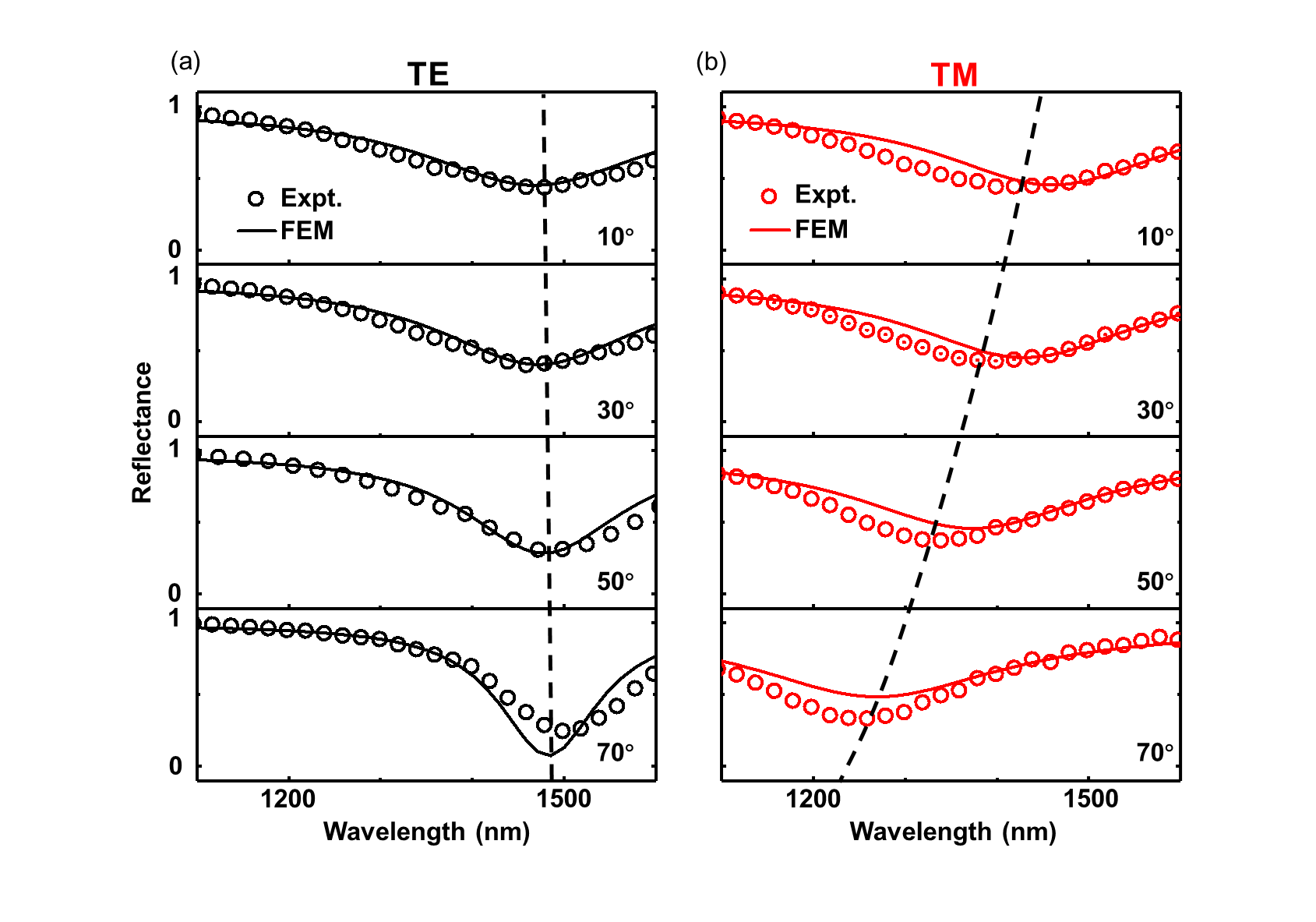


Figure S14. Measured and FEM-simulated reflectance spectra of the angle-multiplexed meta-polarizer in NIR regime shined by (a) TE and (b) TM-polarized light at different incident angles.


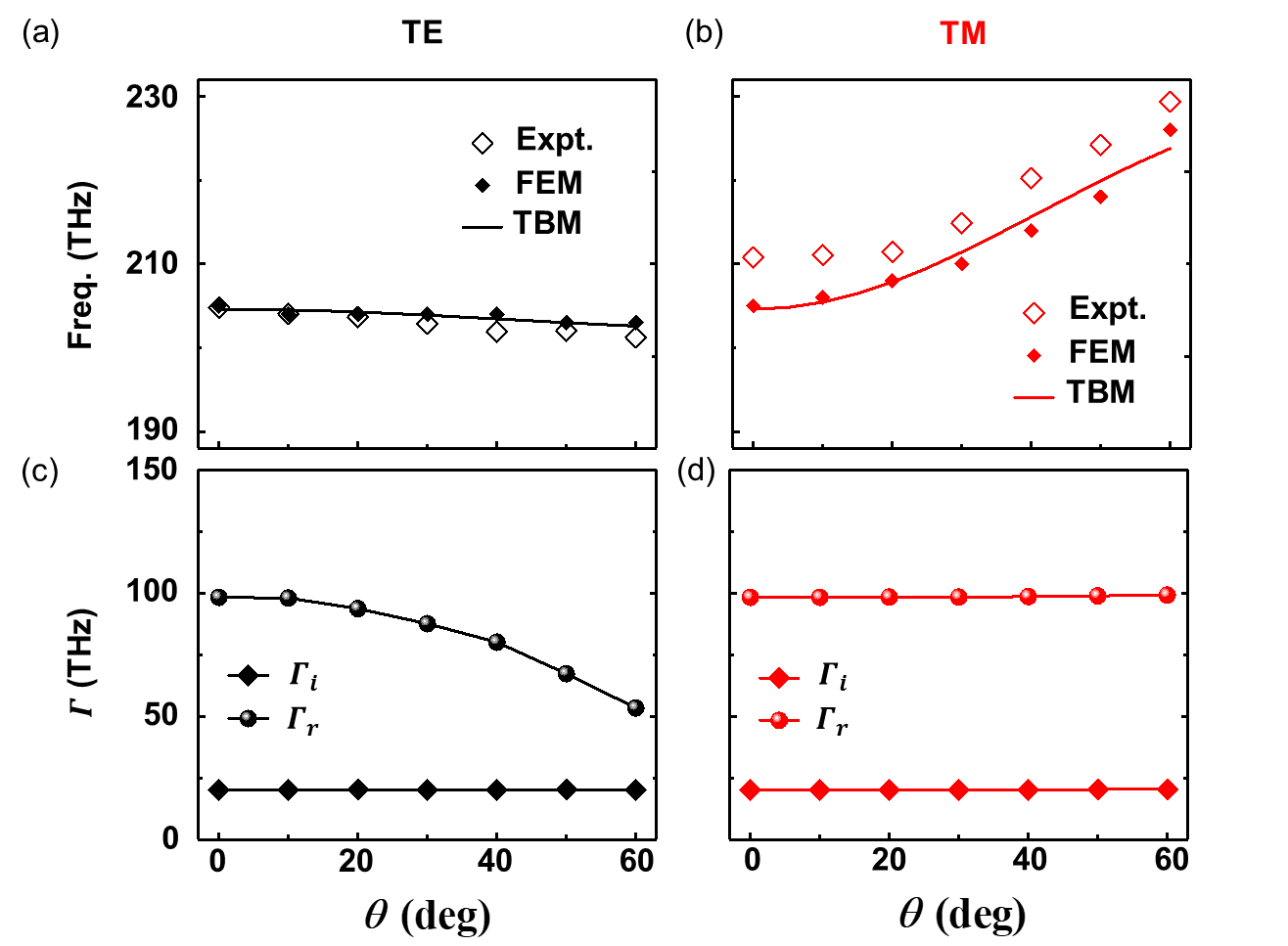


Figure S15. Resonant frequency of the angle-multiplexed meta-polarizer as a function of incident angle, obtained by experiments, FEM simulations and TBM calculations for (a) TE and (b) TM polarized light. Damping parameters $\Gamma_{i}$ and $\Gamma_{r}$ of the meta-polarizer as functions of the incident angle, retrieved from FEM simulations for (c) TE and (d) TM polarized light.

Figure S15 (a-b) compare the $f_{r}\sim\theta$curves of the meta-polarizer in two polarizations, obtained by measurements, FEM simulations and TBM calculations. We find that the TBM results are in reasonable agreement with experimental and numerical ones. In addition, $\Gamma_{i}\sim\theta$and $\Gamma_{r}\sim\theta$relations of the device in both TE and TM polarizations, retrieved from FEM simulations, are also consistent with our theoretical expectations, as shown in Figs. S15(c) and (d).


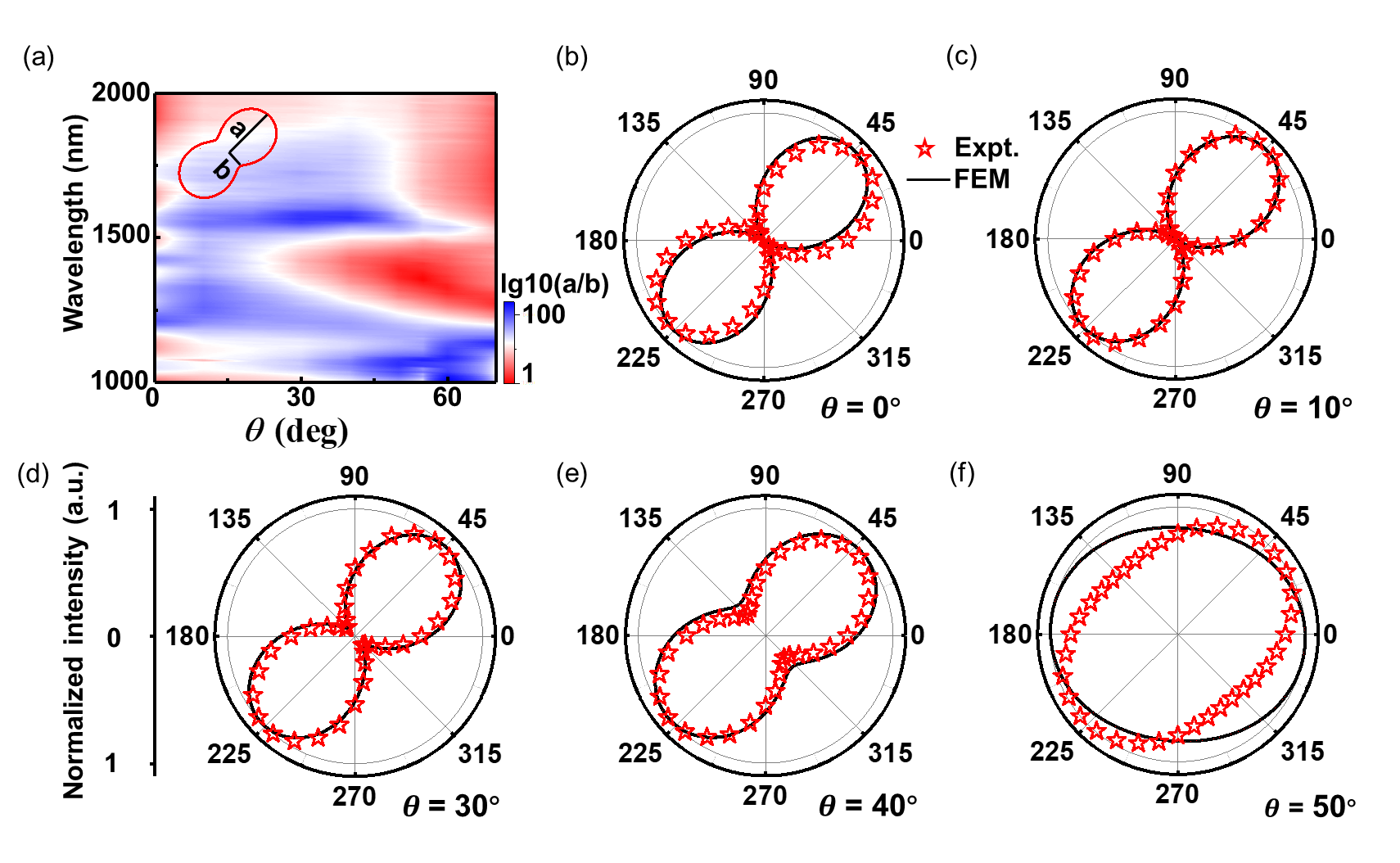


Figure S16. (a) Measured polarization state (*a / b*) versus the wavelength and incident angle with *a* and *b* being the maximum and minimum of the power pattern, respectively. (b)-(f) Measured and simulated normalized power patterns of the metasurface-reflected light filtered by a linear polarizer with rotating polarization direction under different excitation angles of $0^{\circ},$ $10^{\circ},$ $30^{\circ},$ $40^{\circ}$ and $50^{\circ}.$

We experimentally evaluated the absolute working efficiency of the meta-polarizer (studied in Fig. 5 of the main text) at the wavelength 1358 nm and at two different excitation angles $\theta=55^{\circ}$ and $\theta=70^{\circ}$. We first employed the technique described in the main text to measure the patterns for the light beams reflected by our device at two different excitation angles (see Fig. S17a, see also Fig. 5e in the main text), and then repeated the same procedures to obtain the patterns for light beams reflected by a 150nm-thick Ag mirror under the same excitation configuration (see Fig. S17b). For any of the above two incidence angles, we can always carefully analyze the corresponding patterns depicted in Fig. S17a and Fig. S17b to obtain the light powers carried by two orthogonally polarized modes and then obtain the total powers of light beams reflected by the device and the mirror. The ratio between the two values is then the absolute working efficiency of our device. The absolute working efficiencies of our device are 56.6% and 68.6% for $\theta=55^{\circ}$ and $\theta=70^{\circ}$, respectively.


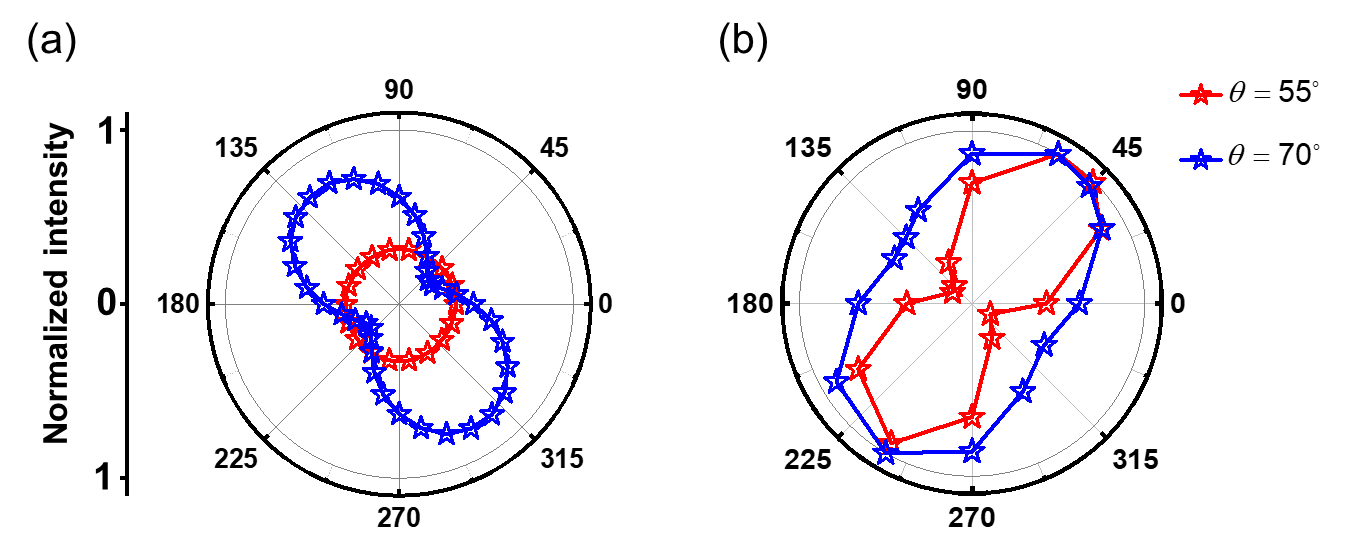


Figure S17. Experimental analysis of polarization state for reflected light from (a) NIR angle-multiplexed meta-polarizer and (b) 150nm Ag film under $\theta=55^{\circ}$ and $\theta=70^{\circ}$ at 1358 nm.

The absolute working efficiency of the device can in principle be further improved by reducing metallic losses in the system, which can be achieved by depositing Ag films with higher qualities. To demonstrate this effect, here we re-performed the same FEM simulations, only with the damping parameter of Ag changing from $\gamma=4.08\times10^{13}s^{-1}$ to $\gamma=1.36\times10^{13}s^{-1}$. When the meta-device is shined by a linearly polarized light with tangential E vector lying at an angle $45^{\circ}$with respect to the *x* axis, the working efficiency ($\eta$) of the device can be numerically evaluated with $\eta=\left(R_{TE}+R_{TM}\right)/2$, with $R_{TE}$ and $R_{TE}$denoting the reflectance for TM and TE polarized light, respectively. Figure S18 depicts the calculated reflectance spectra of our meta-polarizer for TE and TM excitations, and the TE-TM reflection-phase difference as a function of wavelength and incident angle. Comparing to the performance of the meta-device studied in main text, the reduced metallic loss significantly helps improve the absolute working efficiency of the model device at different incident angles. For example, at incidence angle $\theta=55^{\circ}$, the simulated absolute working efficiency is improved to 70.6% (from 57.5%) with DOCP of 98.9% (from 83.7%). At incident angle $\theta=70^{\circ}$, the absolute efficiency of simulated PCR of $90^{\circ}$-polarization rotation is improved to 78.7% (from 69%) at 1400nm.


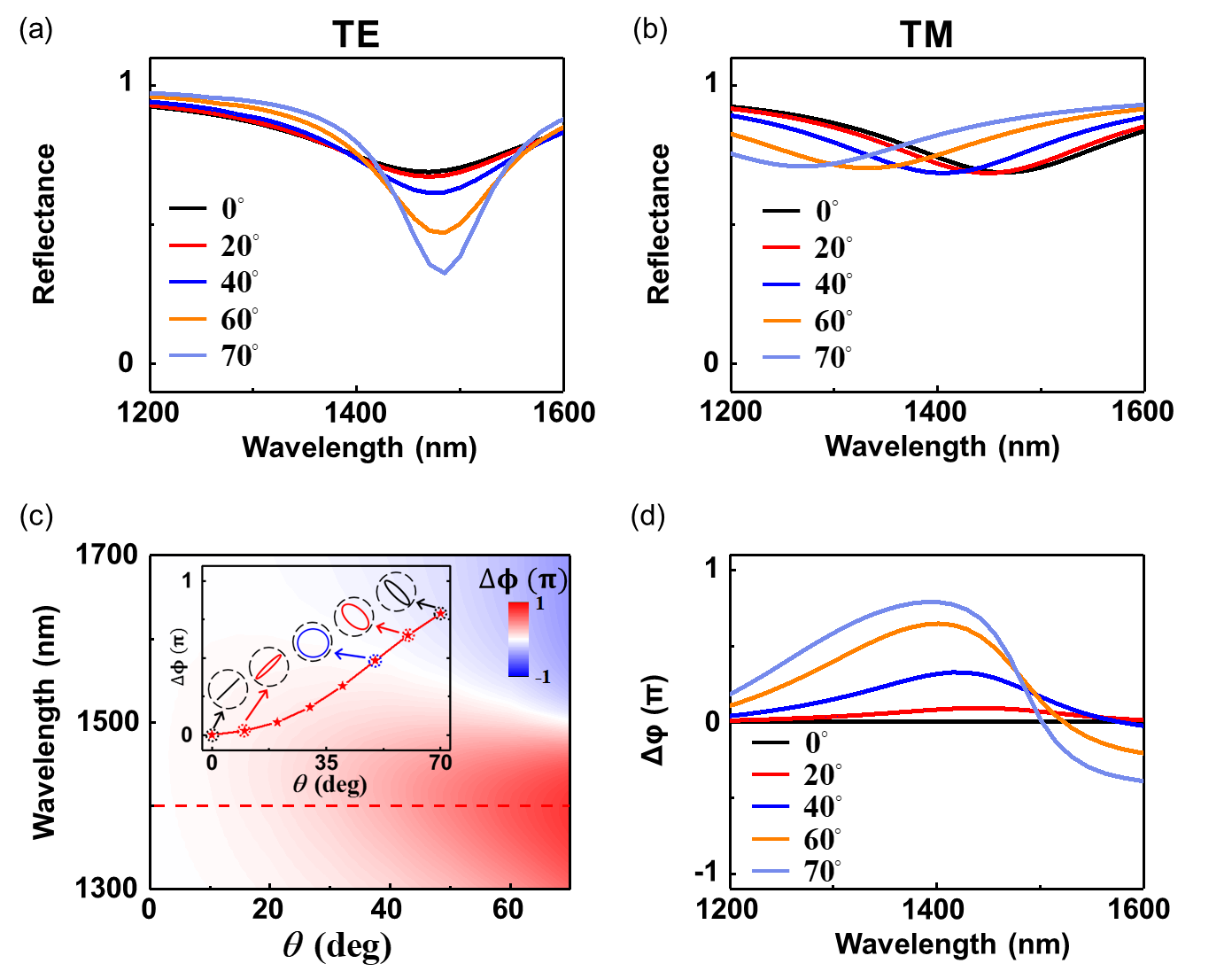


Figure S18. FEM simulated reflectance spectra for (a) TE and (b) TM polarized light of designed angle-multiplexed meta-polarizer with reduced metallic loss under different incident angle of $\theta=0^{\circ},\text{ }20^{\circ},\text{ }40^{\circ},\text{ }60^{\circ},\text{ }70^{\circ}$. (c) FEM-simulated reflection-phase difference between TE and TM polarizations as a function of wavelength and incident angle. Inset: TE-TM reflection phase difference versus incident angle at working wavelength of 1400nm (corresponding to red dashed line), indicating that the reflected beam can exhibit different polarization states. (d) FEM simulated TE-TM reflection phase difference at different incident angles.

**Section 9: Additional simulation data for Fig. 6 in the main text**


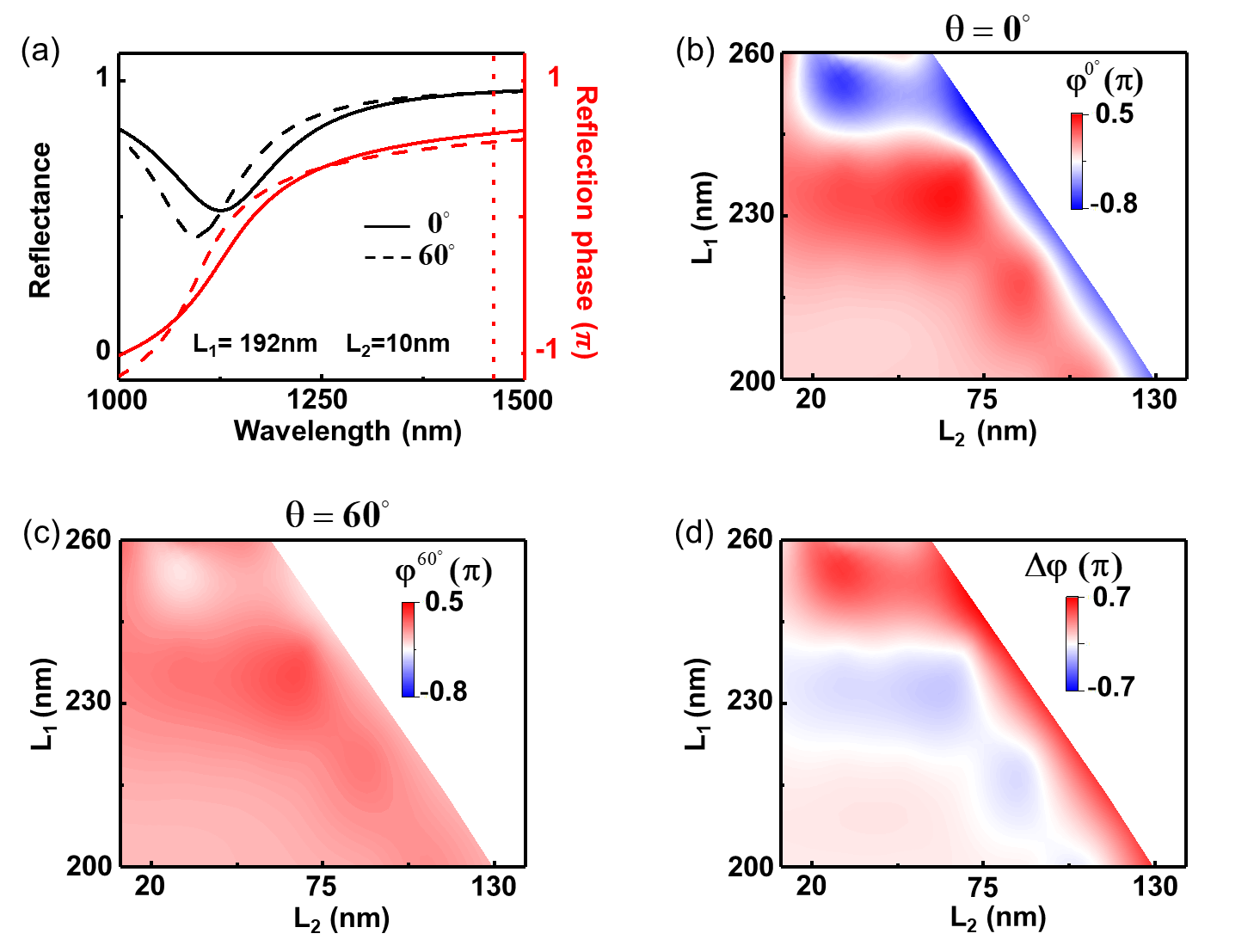


Figure S19. (a) FDTD simulated reflectance and reflection phase spectra of a specific meta-chain ($L_{1}=192nm,\text{ }L_{2}=10nm$) for TM polarized light under incident angles of $0^{\circ}$and $60^{\circ}.$ FDTD simulated reflection phase diagram at (b) $\theta=0^{\circ}$ and (c) $\theta=60^{\circ}$ and (d) reflection phase difference between $\theta=0^{\circ}$ and $\theta=60^{\circ}$ of the Meta-chains with different L_1_, L_2_ for TM polarized light at working wavelength of 1462 nm.

Comparing Fig. S19a with Fig. 6b, we find that we can mainly tune L_1_ to modulate the initial reflection phase at $\theta=0^{\circ}$under TM polarization. Comparing Fig. S19(a) with Fig. 6c, we find that we can mainly tune L_2_ to modulate the couplings between two resonators, which in turn, controls the angular dispersions of such composite meta-atoms to change the phase difference between $\theta=0^{\circ}$ and $\theta=60^{\circ}$at the working wavelength of 1462 nm under TM polarization. Therefore, we get the guideline to design our angle multiplexed wave-front-controller and we can use FDTD simulation to sort out a series of specific meta-atoms satisfying Eq. (6) in the main text.


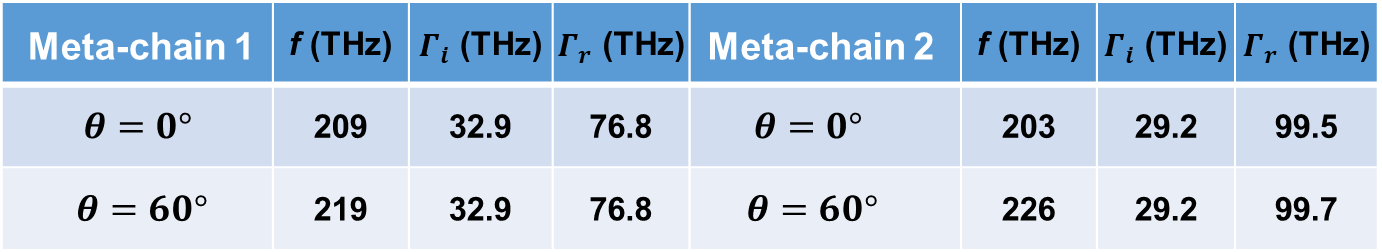


Table S1: Resonant frequencies and damping parameters $\Gamma_{i}$, $\Gamma_{r}$ of the meta-chains studied in Fig. 6 of the main text, at two different incident angles.

Table S1 contains the simulated resonant frequency and the retrieved damping parameters $\Gamma_{i}$, $\Gamma_{r}$ of two specific meta-chains studied in Fig. 6 of the main text, at two incident angles ($\theta=0^{\circ}$ and $\theta=60^{\circ}$). These data clearly show that we can mainly tune L_2_ to modulate the couplings between two resonators, which in turn, controls the resonant frequency shift of such composite meta-atoms.


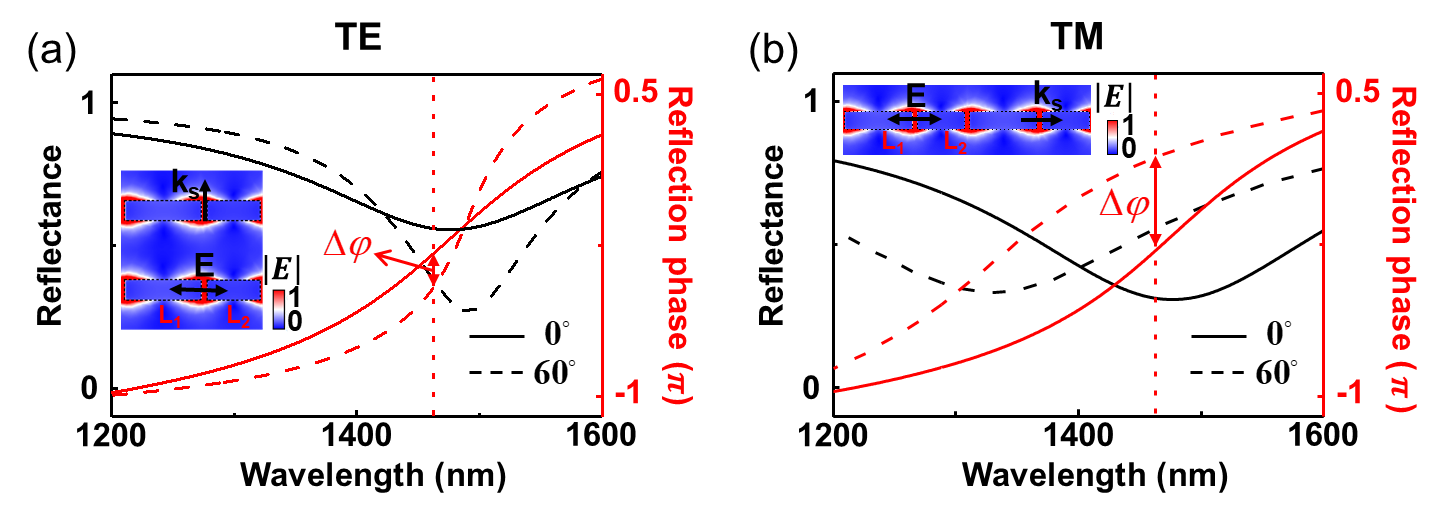


Figure S20. FDTD simulated reflectance and reflection phase spectra of a specific meta-chain under the incident angle $\theta=0^{\circ}$ and $60^{\circ}$for (a) TE and (b) TM polarization. Insets: FDTD-simulated $\left|\vec{E}\right|$ field distributions on the surface of the meta-atom (with *L*_1_ = 192 nm, *L*_2_ = 135 nm) for (a) TE and (b) TM polarization.

The inset in Fig. S20a illustrates that the hot spots in two nearest neighbor meta-atoms are well separated, resulting in very limited contributions to the near-field coupling for the TE case. This fact demonstrates that the couplings between adjacent meta-chains is very weak. Whereas, the $\left|\vec{E}\right|$ field of the resonance modes strongly overlap with each other for TM polarized light (see inset in Fig. S20b), resulting in a much stronger coupling strength in the meta-chain. Therefore we can neglect the near field couplings between meta-chains and utilize periodic metasurface composed of meta-chains to tune geometrical parameters of the meta-chain to satisfy Eq. (6) in the main text.


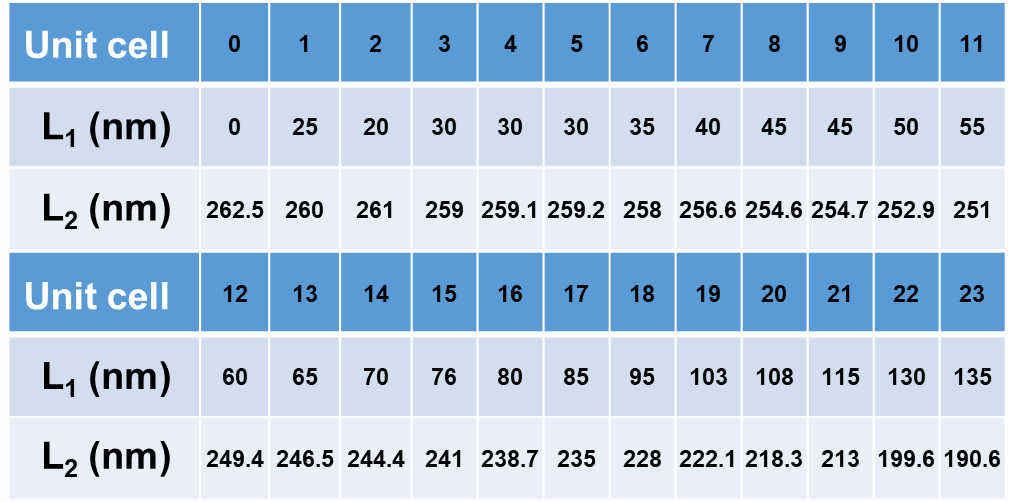


Table S2: Geometrical parameters L_1_, L_2_ of the meta-chains at different positions along *y* direction for the designed angle-multiplexed wave-front-controller.

**References**

1. Qiu M, Jia M, Ma S, Sun S, He Q, Zhou L. Angular Dispersions in Terahertz Metasurfaces: Physics and Applications. *Physical Review Applied* 2018; **9**: 054050.

2. Xi B, Xu H, Xiao S, Zhou L. Theory of coupling in dispersive photonic systems. *Physical Review B* 2011; **83**: 165115.

3. Jackson JD. *Classical Electrodynamics*. John Wiley & Sons, 1999.
